# CircMYH9 drives colorectal cancer growth by regulating serine metabolism and redox homeostasis in a p53-dependent manner

**DOI:** 10.1186/s12943-021-01412-9

**Published:** 2021-09-08

**Authors:** Xin Liu, Yunze Liu, Zhao Liu, Changwei Lin, Fanchao Meng, Lei Xu, Xiuzhong Zhang, Chong Zhang, Penbo Zhang, Shuai Gong, Nai Wu, Zeqiang Ren, Jun Song, Yi Zhang

**Affiliations:** 1 Department of Endocrinology, Affiliated Hospital of Xuzhou Medical University, Xuzhou, 221000 China; 2 Institute of Digestive Diseases, Xuzhou Medical University, Xuzhou, 221002 China; 3 Department of Traditional Chinese Medicine, Affiliated Hospital of Xuzhou Medical University, Xuzhou, 221000 China; 4 Department of General Surgery, Affiliated Hospital of Xuzhou Medical University, Xuzhou, 221000 China; 5 Department of Gastrointestinal Surgery, The Third XiangYa Hospital of Central South University, Changsha, 410013 China

**Keywords:** circMYH9, p53, Colorectal cancer, Serine/glycine metabolism, Redox homeostasis

## Abstract

**Background:**

Circular RNAs (circRNAs) play important roles in cancer progression and metabolism regulation. Serine/glycine metabolism supports the growth of cancer cells by contributing to their anabolic demands and epigenome as well as by regulating their redox state. However, the role of circRNA in the regulation of serine/glycine metabolism has not been well elucidated.

**Methods:**

Microarray analysis was used to screen differentially expressed novel circRNAs. qRT-PCR and FISH were utilized to analyzed the expression of circMYH9. CCK8, colony formation and FACS were used to analyze proliferation of colorectal cancer (CRC) cells. Xenograft experiments were used to analyze tumor growth in vivo. RNA-sequencing, immunoblot and LC–MS were used to identify the downstream metabolic pathway of circMYH9. ChIRP, Mass Spectrometry, RIP and RNA pulldown were utilized to test the interaction between circMYH9, hnRNPA2B1 and p53 pre-mRNA. ChIP-qPCR was used to analyze the binding sites of HIF-1α. Chemically-induced CRC mice were generated to evaluate the role of circMYH9 in tumorigenesis.

**Results:**

We identified an intron-derived circRNA, circMYH9, which was significantly upregulated in CRC tissues. A higher circMYH9 level correlated with shorter relapse-free survival and overall survival of CRC patients. CircMYH9 promoted serine/glycine metabolism, the NAD + /NADH ratio, and glutathione recycling and inhibited reactive oxygen species (ROS) in a p53-dependent manner, impacting tumour growth. Mechanistically, circMYH9 destabilized the pre-mRNA of p53 by recruiting hnRNPA2B1 in the nucleus. hnRNPA2B1 bound to N6-methyladenosine sites on the 3' untranslated region of p53 pre-mRNA and maintained its stability. Moreover, a lack of amino acids led to an elevated level of ROS, resulting in increased HIF1α, which promoted circMYH9 expression by binding to the promoter region. Furthermore, in vivo AAV9-mediated transfection of circMYH9 could drive chemically-induced carcinogenesis by suppressing p53 in mice.

**Conclusions:**

The overexpression of circMYH9 promotes CRC proliferation though modulating serine/glycine metabolism and redox homeostasis in a p53-dependent manner, and targeting circMYH9 and its pathway may be an effective strategy for the treatment of CRC.

**Supplementary Information:**

The online version contains supplementary material available at 10.1186/s12943-021-01412-9.

## Introduction

Metabolic reprogramming is an important characteristic of tumour cells [1]. Tumour cells reprogram their metabolic pathways to meet the material and energy requirements of rapid proliferation [2]. The classic example of metabolic reprogramming in cancer is the Warburg effect or aerobic glycolysis. In addition to glucose metabolism, amino acid metabolism is altered in metabolic reprogramming of cancer cells. Serine and glycine (SG) metabolism, as an important branch of amino acid metabolism, can support cell proliferation [3]. Cells can obtain serine by either import from the extracellular environment or intracellular synthesis from glucose, while the increased demand for serine in tumour cells leads to the reprogramming of serine metabolism [4]. Dysregulated serine metabolism in cancer provides carbons for nucleotide synthesis, increases antioxidant capacity to maintain redox balance, and maintains the stability of the tumour microenvironment [5–8].

CircRNAs are kind of noncoding RNAs (ncRNAs) characterized by covalently closed loops without either polyadenylated tails in the 3' ends or the cap structure at the 5' ends [9]. Dysregulation of circRNAs has been identified in almost all types of cancers and is involved in the tumorigenesis of many tumours, such as colorectal cancer (CRC) [10], breast cancer [11] and liver cancer [12]. Based on their origin, circRNAs are generated from exons or introns of parent genes. Exonic circRNAs account for a major portion of the circRNA family and preferentially localize and function in the cytoplasm, where they can serve as miRNA sponges to affect gene expression or directly bind to proteins to regulate their functions [13]. In addition, circRNAs derived from introns account for a small fraction of all circRNAs; intronic circRNAs have little enrichment for miRNA target sites and are predominantly found in the nucleus to regulate gene expression [14]. However, related studies are lacking, and previous studies have shown that intron-derived circAGO2 physically interacts with the human antigen R (HuR) protein to facilitate its competitive enrichment in the 3'-untranslated region (UTR) of target genes, promoting tumorigenesis and aggressiveness of gastric cancer [15]. These results suggest that intronic circRNAs play crucial roles in cancer progression. However, more functions of intronic circRNAs need to be characterized.

In this study, we identified an intron-derived circMYH9 (hsa_circ_0092283) that was expressed at relatively high levels in CRC cells. CircMYH9 could promote proliferation and serine metabolism in CRC cells. Mechanistically, we demonstrated that circMYH9 increased endogenous serine production by inhibiting the stability of p53 pre-mRNA. Interestingly, a lack of amino acids led to an elevated level of reactive oxygen species (ROS), resulting in increased HIF1α, which promoted circMYH9 expression by binding to the promoter region. The function of circMYH9 in CRC carcinogenesis was verified in mice using adeno-associated virus (AAV) 9-mediated overexpression of circMYH9.

## Materials and methods

### Patient samples

This study was reviewed and approved by the Ethics Committee of the Affiliated Hospital of Xuzhou Medical University (XYFY2019-KL221-01), and written informed consent was obtained from all patients. The study included 148 CRC patients aged 30 to 80 years. All procedures were undertaken in accordance with guidelines set forth by Declaration of Helsinki. Tumor tissues and their adjacent normal tissues were obtained at surgery from patients suffering from CRC. Patients with a history of preoperative chemotherapy or radiotherapy were excluded. All fresh specimens were frozen in liquid nitrogen, validated by pathological diagnosis, and stored at -80 °C until use.

### Cell lines and cell culture

The human normal colonic epithelial cell line FHC and the CRC cell lines SW480, HT-29, HCT8, DLD1, HCT116 and LoVo were purchased from the American Type Culture Collection (ATCC). HCT116 cells were cultured in McCoy’s 5A medium (Hyclone, USA) containing 10% fetal bovine serum (Gibco, USA), 100U/ml penicillin and 100 μg/ml streptomycin (Vicmed, China) while FHC and SW480 cells were cultured in DMEM medium and HT-29, DLD1, HCT8 and LoVo cells were cultured in RPMI 1640 medium. The cell lines were routinely screened for mycoplasma contamination using the Mycoplasma qPCR Detection Kit (Sigma, Shanghai, China). For starvation experiments cells were fed the same media formulation without serine and glycine or without glutamine. All cells were cultured at 37 °C in a humidified incubator with 5% CO_2_.

### Reagents

The following antibodies were used: anti-CDK1 (abcam, 2546 ab133327), anti-cyclin D1 (CST, 2922), anti-CDK6 (Abcam, ab151247), anti-cyclin E2 (CST, 4132), anti-PHGDH (Proteintech, 14,719–1-AP), anti-PSPH (Proteintech, 14,513–1-AP), anti-Ki-67 (Abcam, ab15580), anti-SLC1A4 (Proteintech, 13,067–2-AP), anti-p53(CST, 9282), anti-MDM2 (CST, 86,934), anti-hnRNPA2B1 (Proteintech, 14,813–1-AP), anti-Mettl3 (Proteintech, 15,073–1-AP), anti-GAPDH (CST, 2118), anti-Flag (CST, 2368), HRP goat anti-rabbit IgG (Biodragon, BF03008), HRP goat anti-mouse IgG (Biodragon, BF03001), CoraLite488-conjugated goat anti-rabbit IgG (Proteintech, SA00013-2), CoraLite594-conjugated goat anti-mouse IgG (Proteintech, SA00013-3), Nutlin-3 was purchased from Sigma (Shanghai, China).

### Cell transfection

Short interfering RNA (siRNA) sequences were directly synthesized (GenePharma, Shanghai, China). The siRNAs were transfected into cells using Lipofectamine 3000 (Invitrogen, Shanghai, China). Two days later, the cells were harvested for further experiments.

shRNAs were delivered by lentiviral infection with lentiviruses produced by transfection of 293 T cells with the vector pLKO.1 Cells infected with lentiviruses delivering scrambled shRNA (shScr) were used as negative control cells. For overexpression of circMYH9, Human circMYH9 linear sequence (GenePharma, Shanghai, China) was obtained and inserted into lentiviral vector pLCDH-ciR (Geenseed Biotech Co., Guangzhou, China). For overexpression of Mettl3, Full-length Mettl3 were also synthesized by GENEWIZ (Beijing, China) and cloned into the pcDNA3.1 vector (Invitrogen, Shanghai, China). The shRNA and siRNA sequences are listed in Supplemental Materials and Methods.

### RNA-seq assay

Total RNA of cancer cells (1 × 10^6^) was extracted in accordance with the manual of TRIzol®reagent (Invitrogen, Shanghai, China). Library preparation and transcriptome sequencing on an MGISEQ-2000 platform were carried out at Wuhan Genomics Institute (BGI, Wuhan, China) to generate 100-bp paired-end reads. MGISEQ-2000 was applied in counting the numbers of read mapping to each gene, and fragments per kilobase of transcript per million fragments mapped (FPKM) of each gene were calculated. FC (fold change) was used to describes the difference of gene expression.

### Quantitative real-time PCR (qRT-PCR)

Total RNA was extracted from tissues using TRIzol according to the manufacturer’s protocol. Nuclear and cytoplasmic RNA were isolated from cells using RNA Subcellular Isolation Kit (Active Motif, Shanghai, China). cDNA synthesis was performed using a HiScript Q Select RT SuperMix for qPCR (+ gDNA wiper) (Vazyme Biotech Co., Nanjing, China). qRT-PCR analysis was performed using SYBR Green qPCR SuperMix Kit (Vazyme Biotech Co., Nanjing, China) according to the manufacturer’s protocol. The expression of the target genes was normalized to that of GAPDH. The primers used for amplification are described in Supplemental Materials and Methods.

### Gene set enrichment analysis (GSEA)

GSEA was performed on the normalized data using the GSEA v2.0 tool (http://www.broad.mit.edu/gsea/). We compared the gene expression between cells transfected with control siRNA and circMYH9 siRNA. The detailed genes in gene sets can refer to MSigDB (http://software.broadinstitute.org/gsea/msigdb/genesets.jsp). The *P* values of the differences between the two gene sets were analyzed with the Kolmogorov–Smirnov test.

### Mouse strains and maintenance

p53^flox/flox^ mice were generated by Cyagen Biosciences Inc., (Guangzhou, China). p53^flox/flox^ mice were crossed with Villin-Cre transgenic mice to generate p53 ^−/−^, Villin-Cre mice (p53^KO^). One week before the experiment, AAV9-circMYH9 (GenePharma, Shanghai, China) overexpression and AAV-control were administered by enema. p53^KO^ and p53^WT^ mice were intraperitoneally injected with 12 mg/kg of azoxymethane (AOM; Sigma, Shanghai, China). After 5 days, the mice were treated with 2% dextran sulfate sodium (DSS; MP Biomedicals, Santa Ana, CA) in drinking water for 5 days, which was then followed by 14 days of regular water. This cycle was repeated thrice. On day 60, mice were sacrificed. Polyp load was identified as a sum of the diameters of all tumors in a given mouse. Mouse experiments were performed following general guidelines issued by the Laboratory Animal Care Evaluation and Identification Association.

### Chromatin isolation by RNA purification (ChIRP)

The ChIRP assay was performed using the Magna ChIRP RNA Interactome Kit (Millipore, USA) following the manufacturer’s guidelines. Briefly, a total of 1 × 10^7^ cells was lysed in complete lysis buffer for each reaction, and the DNA was then sheared into small fragments through sonication. Then the lysate was incubated with biotin-labeled probes that could hybridize with circMYH9 or control probe. Finally, the probes were extracted by streptavidin magnetic beads, and the combined protein was isolated for mass spectrometry (MS).

### In vivo tumor growth assay

Six-week-old male BALB/c nude mice were obtained (Shanghai Slac Laboratory Animal Co. Ltd., China) and bred under specific pathogen-free conditions. The HCT116 cell line with stable circMYH9 knocked down or a control HCT116 cell line and circMYH9-overexpressing or control LoVo cells were used for the in vivo tumour growth assay. Cancer cells (5 × 10^6^) subcutaneously injected into the flank regions of the mice (n = 5 per group, calculated by paired t tests). Over a period of 3 weeks, tumor formation in the mice was observed by measuring the tumor volume. Then, the tumors were excised and weighed. All animal experiments were reviewed and approved by Xuzhou Medical University.

### Statistical analysis

The significance of the differences was determined via one-way ANOVA or Student’s t-test. Spearman’s correlation coefficient was used to calculate the correlations between the two groups. Kaplan–Meier analysis was employed for survival analysis, and the differences in the survival probabilities were estimated using the log-rank test. *P* < 0.05 was considered to indicate statistical significance. The statistical analyses were performed using SPSS version 22.0 (SPSS, Inc.).

Immunoblot (IB), fluorescence in situ hybridization (FISH), chromatin immunoprecipitation (ChIP), stable isotope tracing experiments, intracellular ROS, glutathione and NAD + /NADH measurement, colony formation, dual luciferase reporter assay, immunohistochemistry (IHC), Cell Counting Kit-8 (CCK8), and RNA immunoprecipitation (RIP): details of the above assays are provided in the Supplemental Materials and Methods.

## Results

### circMYH9 was identified in CRC tissues and cells

**Fig. 1:**
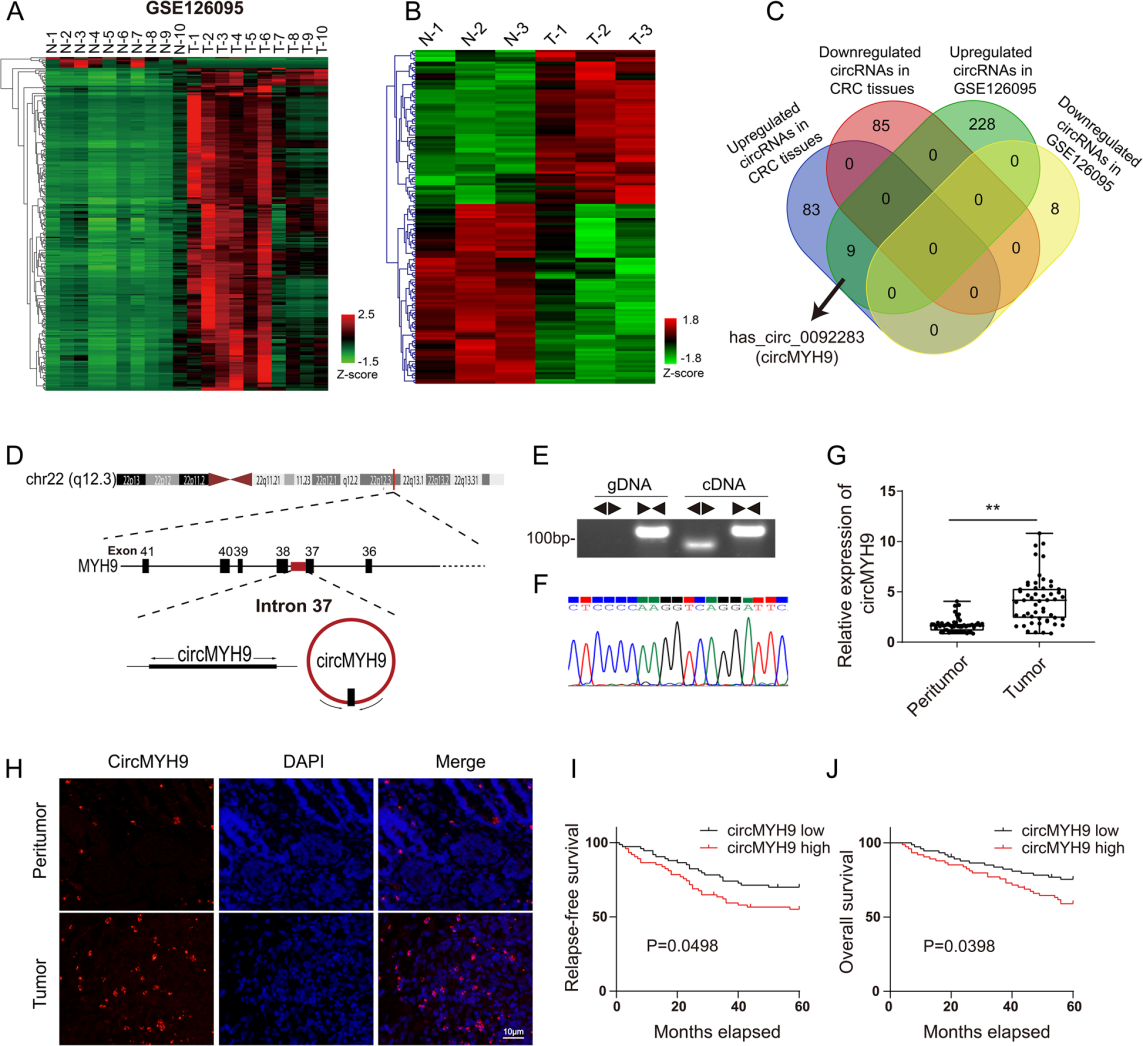
CircMYH9 was upregulated in CRC tissues and predicted poor prognosis. **A-B** Heat maps of differentially expressed circRNAs in CRC tissues and adjacent normal tissues obtained from GSE126095 (A) and 3 CRC patients (B). **C** Venn diagram showing the numbers of overlapping circRNAs between the differentially expressed circRNAs in GSE126095 and 3 CRC patients. **D** Schematic diagram of the genomic location of circMYH9 derived from its parent gene MYH9. **E** The panel shows the detection of circMYH9 using divergent and convergent primers from cDNA or gDNA of HCT116 cells. **F** CircMYH9 was further validated by Sanger sequencing. **G** The relative expression levels of circMYH9 were assessed by qRT-PCR in 53 paired normal tissues and CRC tissues (*, *P* < 0.05; **, *P* < 0.01). **H** Immunofluorescence analysis of circMYH9 expression in peritumour tissues and CRC tissues. **I-J** Kaplan–Meier analysis of the RFS and OS rates in 148 CRC patients with high or low expression of circMYH9

To identify potential differentially expressed circRNAs, we screened circRNAs that may be differentially expressed in 10 paired CRC tissues and adjacent tissues from the GEO database (GSE126095). A total of 8 downregulated circRNAs and 237 upregulated circRNAs (FDR < 0.01, |logFC|≥ 2) were detected (Fig. 1A). Then, we further analysed the circRNA expression profiles in 3 pairs of CRC tissues and adjacent tissues via microarray analysis (the characteristics were shown in Table S1), and 92 circRNAs were upregulated and 85 circRNAs were downregulated (FDR < 0.01, |FC|≥ 2, Fig. 1B). 9 overlapped circRNAs were identified by intersecting 237 upregulated circRNAs in 10 paired CRC tissues with 92 upregulated circRNAs in 3 paired CRC tissues (Fig. 1C, Figure S1A). Among the 9 circRNAs, hsa_circ_0092283, also known as circMYH9, originated from the MYH9 gene and consisted of the head-to-tail splicing of intron 37 (Fig. 1D). Because circMYH9 was significantly upregulated in ten paired (logFC = 3.63) and three paired CRC tissues (FC = 4.51), compared to the other 8 circRNAs (Figure S1A), we selected it for further study.

To validate the cyclization of circMYH9 in CRC cells, we designed two sets of primers for MYH9: convergent primers for the linear form and divergent primers for the circular form. RT-PCR and qRT-PCR analysis indicated that circMYH9 could be amplified by divergent primers in cDNA, and no other products were observed in the genomic DNA (gDNA) groups (Fig. 1E). Furthermore, circMYH9 was resistant to RNase R, while MYH9 mRNA can be degraded by RNase R (Figure S1B). Then, the RT-PCR product of circMYH9 was confirmed by Sanger sequencing (Fig. 1F).

### CircMYH9 expression was upregulated in CRC tissues and predicted poor prognosis

We analysed the circMYH9 expression levels in CRC tissues, and qRT-PCR and FISH revealed that circMYH9 expression was higher in the CRC tissues than in the peritumoral tissues (Fig. 1G, H, Figure S1C). Then, we examined the correlation of circMYH9 expression with clinicopathological findings in 148 CRC cases. The patients were stratified into 2 groups based on the median circMYH9 expression levels detected by qRT-PCR. The circMYH9 levels were significantly associated with tumour size, distant metastasis, lymph node metastasis, TNM stage and p53 status (Table 1). We examined the correlation between circMYH9 expression and data from the five-year follow-up of the patients. Using the median expression value of circMYH9 in 148 patients as the cut-off point, we performed Kaplan–Meier analysis, which revealed that the patients with low expression of circMYH9 had a higher relapse-free survival (RFS) rate than those with high expression (Fig. 1I), the hazard ratio was 0.593 (95% CI, 0.366–0.997). Furthermore, the patients with high expression of circMYH9 had a significantly lower overall survival (OS) rate than patients with low expression of circMYH9 (Fig. 1J), the hazard ratio was 0.547 (95% CI, 0.305–0.982).

**Table 1:** Relationships between circMYH9 expression and the clinicopathological characteristics of CRC patients

**Variables**	**Cases**	**circMYH9 expression**		***P* value**	χ^2^
		**Low(n = 74)**	**High(n = 74)**		
**Age (years)**					
≤ 62 years	91	46	45	0.866	2.244
> 62 years	57	28	29		
**Gender**					
Female	71	39	32	0.249	0.101
Male	77	35	42		
**Tumor size**					
≤ 5.0 cm	76	30	46	0.009^**^	0.000
> 5.0 cm	72	44	28		
**Distant metastasis**					
Negative	129	69	60	0.027^*^	0.001
Positive	19	5	14		
**Differentiation**					
Poor	59	26	33	0.240	0.093
Well to moderate	89	48	41		
**Lymph node metastasis**					
Negative	76	45	31	0.021^*^	0.001
Positive	72	29	43		
**TNM stage**					
I–II	78	46	32	0.021^*^	0.001
III–IV	70	28	42		
**MSI status**					
MSI-H	11	4	7		
MSI-L/MSS	137	70	69	0.347	0.202
**p53 status**					
Negative	60	23	37		
Positive	88	51	37	0.019^*^	0.001

^*^P < 0.05, **P < 0.01

### CircMYH9 promoted cell growth in CRC cells

**Fig. 2:**
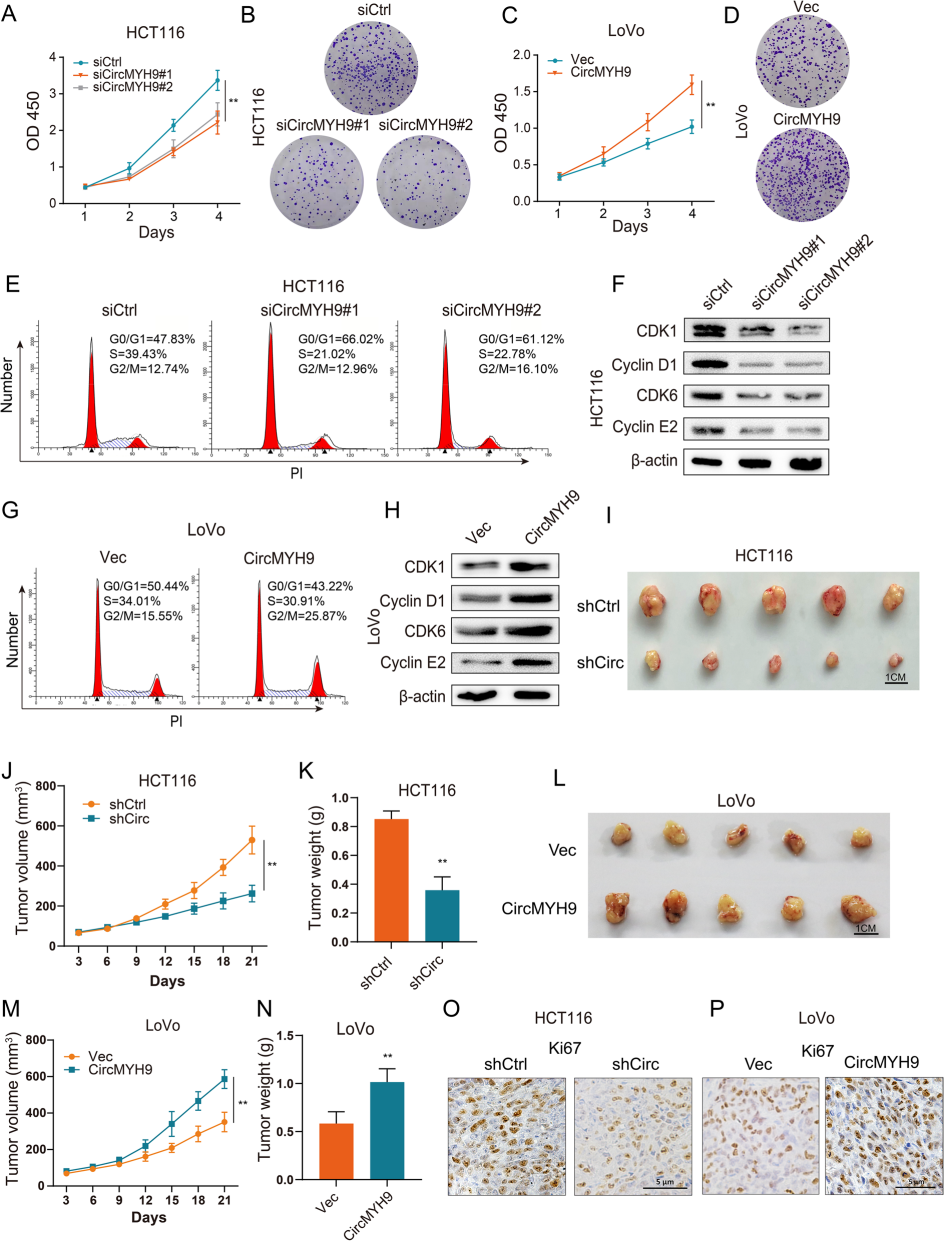
CircMYH9 promoted cell growth in CRC cells. **A-B** CCK-8 and colony formation assays detected cell viability of the circMYH9-depleted HCT116 cells and control HCT116 cells. **C-D** CCK-8 and colony formation assays detected cell viability of the circMYH9-overexpressing LoVo cells and control LoVo cells. **E** Flow cytometry was used to assess the cell cycle of the circMYH9-depleted HCT116 cells and control HCT116 cells. **F** Cell cycle proteins (CDK1, Cyclin D1, CDK6, Cyclin E2) were assessed by immunoblotting in the circMYH9-depleted HCT116 cells and control HCT116 cells. **G** Flow cytometry was used to assess the cell cycle of circMYH9-overexpressing LoVo cells and control LoVo cells. **H** Cell cycle proteins (CDK1, Cyclin D1, CDK6, Cyclin E2) were assessed by immunoblotting in the circMYH9-overexpressing LoVo cells and control LoVo cells. **I** Image of xenograft tumours in BALB/c nude mice subcutaneously injected with the circMYH9-depleted and their respective parental cells (5 × 10^6^ cells per mouse). The mice were sacrificed after 3 weeks, and subcutaneous xenograft tumours were collected. **J** Tumour growth curves represented as the tumour volume of the circMYH9-depleted HCT116 cells and their respective parental cells in xenograft models at the indicated time intervals (n = 5 per group). **K** Tumour weights of the two groups were measured after sacrifice (n = 5 per group). **L-N** Image of xenograft tumours, tumour growth curves and tumour weights in BALB/c nude mice subcutaneously injected with the circMYH9-overexpressing and their respective parental cells (5 × 10^6^ cells per mouse). **O-P** IHC analysis of Ki-67 expression in sections obtained from xenograft models. Data are shown as the mean ± SD from three independent experiments (*, *P* < 0.05; **, *P* < 0.01)

To explore the potential role of circMYH9 in CRC cells. We first examined its expression in normal intestinal epithelial cell line FHC and CRC cell lines and found that the expression of circMYH9 was remarkedly upregulated in CRC cell lines compared with FHC cells (Figure S1D). Then we assessed the effects of circMYH9 on CRC proliferation. CCK-8 and colony formation assays demonstrated that circMYH9 knockdown resulted in significant inhibition of tumour growth in HCT116 and HCT8 cells (Fig. 2A, B, Figure S1E-H). In contrast, circMYH9 overexpression increased LoVo cell growth (Fig. 2C, D, Figure S1I, J). Intriguingly, compared to the above-mentioned p53 wild type (wt) CRC cell lines, circMYH9 knockdown only slightly altered cell proliferation in p53 mutated DLD1 and HT-29 cell lines (Figure S1K, L). Cell cycle distribution analysis showed that circMYH9 knockdown led to arrest of the cell cycle at the G0/G1 phase, with a corresponding reduction in the percentage of cells in the S and G2/M phases (Fig. 2E, Figure S1M-O). Cell cycle proteins (CDK1, Cyclin D1, CDK6, Cyclin E2) were significantly reduced upon circMYH9 knockdown (Fig. 2F, Figure S1P), whereas cell cycle was promoted and cell cycle proteins were increased after circMYH9 overexpression in CRC cells (Fig. 2G, H, Figure S1Q). As HCT116 and LoVo cell lines were relatively higher and lower respectively in p53 wt CRC cell lines, we chose them for further study.

We next examined the effect of circMYH9 on CRC tumour growth in vivo. HCT116 cells stably transfected with circMYH9 shRNA or shCtrl control and LoVo cells stably transfected with circMYH9 plasmid or vector control were subcutaneously injected into nude mice, the tumour volume was measured after injection for 21 days, and the tumour weight was measured after sacrifice. CircMYH9 depletion significantly suppressed tumour volume and weight compared to those of shCtrl control cells (Fig. 2I-K), in contrast, CircMYH9 overexpression exhibited increased tumour volume and weight compared to those of vector control cells (Fig. 2L-N). IHC analysis showed that the tumours formed from the circMYH9 shRNA-transfected HCT116 cells exhibited weaker Ki67 staining (Fig. 2O, Figure S1R) and that formed from circMYH9 overexpressing LoVo cells exhibited stronger Ki67 staining than the tumours derived from the vector-transfected cells (Fig. 2P, Figure S1S).

### CircMYH9 promoted SG metabolism though p53-mediated upregulation of PHGDH

**Fig. 3:**
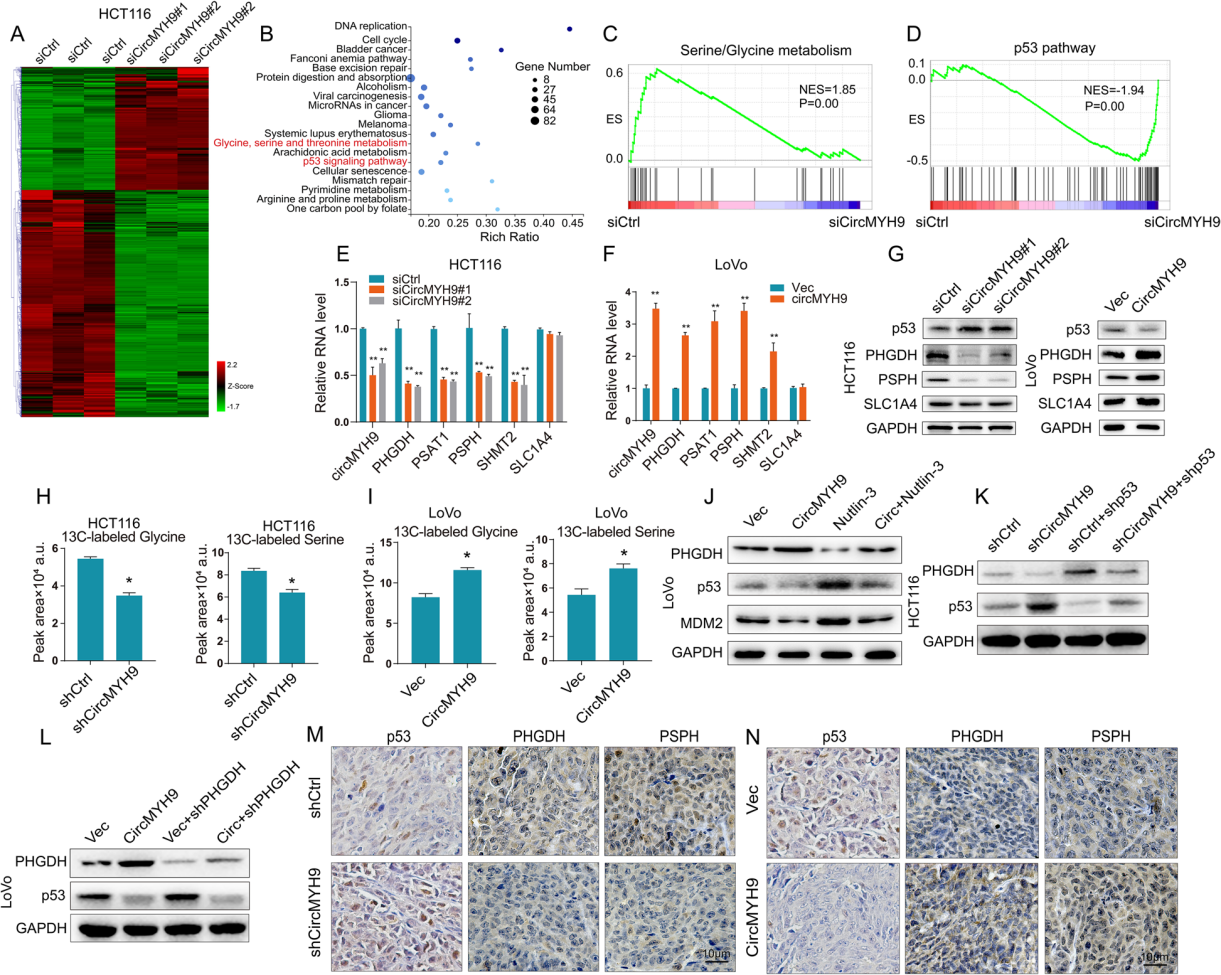
CircMYH9 promoted SG metabolism though p53-mediated upregulation of PHGDH. **A** Heatmap of the differentially expressed genes after circMYH9 knockdown in HCT116 cells. **B** Bubble chart showing the GO analysis of the differentially expressed genes after circMYH9 knockdown. **C-D** GSEA showed that genes differentially expressed in response to circMYH9 knockdown were enriched in gene sets of SG metabolism and p53 pathway. **E–F** SG biosynthetic pathway genes and the neutral amino acid transporter SLC1A4 were detected by qRT-PCR in circMYH9-depleted or circMYH9-overexpressing CRC cells. **G** The expression of p53, PHGDH, PSPH and SLC1A4 were detected by IB in circMYH9-depleted or circMYH9-overexpressing CRC cells. **H-I** HCT116 cells expressing a control or circMYH9-shRNA and LoVo cells expressing vector or circMYH9 plasmid were cultured in complete medium with uniformly labelled [U-13C] glucose for 24 h. LC–MS was used to detect the relative intracellular levels of 13C-labelled serine or glycine. The mean value of the peak area ± SD represents the serine and glycine peaks on the MS chromatogram from 5 times of independent experiments(*, *P* < 0.05; **, *P* < 0.01). **J** IB analysis of PHGDH, p53 and MDM2 expression in the LoVo cells treated with circMYH9 overexpression plasmid or/and Nutlin-3. **K** IB analysis of PHGDH and p53 expression in the circMYH9-depleted HCT116 cells treated with shRNA ctrl or p53 shRNA. **L** IB analysis of PHGDH and p53 expression in the circMYH9-overexpressing LoVo cells treated with shRNA ctrl or PHGDH shRNA. **M** IHC staining of p53, PHGDH and PSPH in xenograft tumours transfected with control or circMYH9-depleted HCT116 cells. **N** IHC staining of p53, PHGDH and PSPH in xenograft tumours transfected with vector or circMYH9-overexpressing LoVo cells. Data (except **H** and **I**) are shown as the mean ± SD of three independent experiments (*, *P* < 0.05; **, *P* < 0.01)

To gain insight into the molecular mechanism by which circMYH9 promotes colorectal tumorigenesis, we performed RNA-seq in both control and circMYH9-depleted HCT116 cells and identified differentially expressed genes. CircMYH9 depletion resulted in upregulation of 893 genes and downregulation of 1650 genes (|FC|≥ 2, *p* < 0.01, Fig. 3A). Then, functional enrichment analysis was conducted using gene ontology (GO) analysis and GSEA analysis. The top 20 GO terms of biological process are presented in Fig. 3B, including glycine, serine and threonine metabolism and p53 signaling pathway. GSEA results showed that the differentially expressed gene sets were significantly related to SG metabolism and p53 pathway (Fig. 3C, D, Figure S2A, B). Recent studies have also reported that SG synthesis can support tumour growth [16]. We silenced or overexpressed circMYH9 and found that it positively regulated the expression of SG biosynthetic pathway genes (PHGDH, PSAT1, PSPH and SHMT2) (Fig. 3E-G). Intracellular levels of serine and glycine are maintained through uptake of exogenous pools and by de novo synthesis from the glycolytic intermediate 3-phosphoglycerate (3PG) [17]. Silencing or ectopic expression of circMYH9 hardly altered the level of the neutral amino acid transporter SLC1A4 (Fig. 3E-G), suggesting that circMYH9 cannot affect the uptake of SG. We further evaluated whether circMYH9 controls SG levels via glycolysis. HCT116 cells expressing control or circMYH9-shRNA and LoVo cells expressing vector or circMYH9 plasmid were incubated with uniformly labelled [U-13C] glucose for the last hour of culture, and 13C-labelled intracellular metabolites were analysed by liquid chromatography-mass spectrometry (LC–MS). The results showed a significant decrease in SG in the shRNA-transfected HCT116 cells relative to the control cells and a significant increase in SG in circMYH9-overexpressing LoVo cells relative to the vector cells (Fig. 3H, I).

Because p53 suppresses PHGDH expression and is closely associated with de novo serine biosynthesis in melanomas [18], we evaluated whether circMYH9 regulated the de novo synthesis of SG though the p53 pathway. qRT-PCR and IB demonstrated the negative regulation of p53 by circMYH9 (Fig. 3G, Figure S2C, E). Nutlin-3 is a nongenotoxic drug that activates p53 by interrupting the Mdm2-p53 interaction. Nutlin-3 treatment in the circMYH9-overexpressing LoVo cells reversed the downregulation of p53 and MDM2 and suppressed SG biosynthetic pathway gene expression and cell proliferation (Fig. 3J, Figure S2C, D). while, p53 silencing in the circMYH9-depleted HCT116 cells showed the opposite effects (Fig. 3K, Figure S2E, F). We also knockdown PHGDH with a shRNA in the circMYH9-overexpressing LoVo cells. PHGDH suppression showed similar effects as Nutlin-3 treatment without affecting p53 expression (Fig. 3L, Figure S2G, H). Moreover, IHC of in vivo xenografts with circMYH9 depletion displayed higher expression of p53 and lower expression of the PHGDH and PSPH than those of the control cells (Fig. 3M, Figure S2I). In contrast, IHC of circMYH9 overexpressing xenografts showed the lower expression of p53 and higher expression of the PHGDH and PSPH than those of the vector cells (Fig. 3N, Figure S2J). These results confirmed the major role of circMYH9 in the regulation of p53 pathway and de novo SG metabolism.

To investigate the mechanisms by which p53 suppresses PHGDH, we cotransfected a pGL3-PHGDH promoter-luciferase construct plasmid into CRC cells with p53 shRNA or nutlin-3. We found that luciferase activities were significantly increased in p53-depleted LoVo cells and decreased in nutlin-3 treated HCT116 cells (Figure S2K, L). Then we further verified whether circMYH9 promotes PHGDH transcription using PHGDH promoter-luciferase reporter. As expected, circMYH9 knockdown significantly reduced the PHGDH luciferase activity in HCT116 cells (Figure S2M), whereas circMYH9 overexpression enhanced the PHGDH luciferase activity in LoVo cells (Figure S2N). A previous study reported that the -606/-633 region in the PHGDH promoter was identified as the p53 binding site [18]. We performed ChIP using an anti-p53 antibody and amplified the p53-bound DNA fragments using primers flanking the p53 binding sites. High enrichment of p53 at the reported binding sites was observed, furthermore, the occupancy of p53 was decreased upon p53 knockdown and increased upon nutlin-3 treatment (Figure S2O, P). These results suggested that PHGDH is transcriptionally suppressed by p53.

### CircMYH9 maintained redox homeostasis in a p53-dependent manner

As SG metabolism contributes to the maintenance of redox status, and we investigated whether circMYH9 affected redox status. Glutathione (GSH) production, the NAD + /NADH ratio, and the ROS levels were evaluated. CircMYH9 depletion resulted in increased ROS levels (Figure S3A), whereas ectopic expression of circMYH9 reduced ROS in CRC cells (Figure S3A). We also used flow cytometry and immunofluorescence to measure the ROS level in CRC cells. CircMYH9 depletion increased ROS levels, whereas ectopic expression of circMYH9 showed the opposite results (Figure S3B, C). Furthermore, the glutathione/glutathione disulphide (GSH/GSSG) ratio was decreased upon circMYH9 knockdown and increased upon circMHY9 overexpression (Figure S3D). We also measured the NAD + /NADH ratio after circMYH9 knockdown. CircMYH9 suppression caused a significant reduction in the NAD + /NADH ratio in HCT116 cells (Figure S3E). Next, we evaluated whether circMYH9 maintains redox homeostasis though the p53 pathway. The decreased ROS levels in the circMYH9-overexpressing HCT116 cells were partly rescued by treatment with Nutlin-3 or shPHGDH (Figure S3F). These results suggested that circMYH9 regulates redox homeostasis via p53 and its target PHGDH. Moreover, we evaluated whether the role of circMYH8 in redox homeostasis influenced cell growth. As expected, we found that the ROS scavenger N-acetylcysteine (NAC) partly restored the proliferation of circMYH9-depleted HCT116 cells (Figure S3G). These data suggested that the p53-dependent role of circMYH9 in redox status is important for the growth of CRC cells.

### CircMYH9 regulated p53 pre-mRNA stability through hnRNPA2B1

**Fig. 4:**
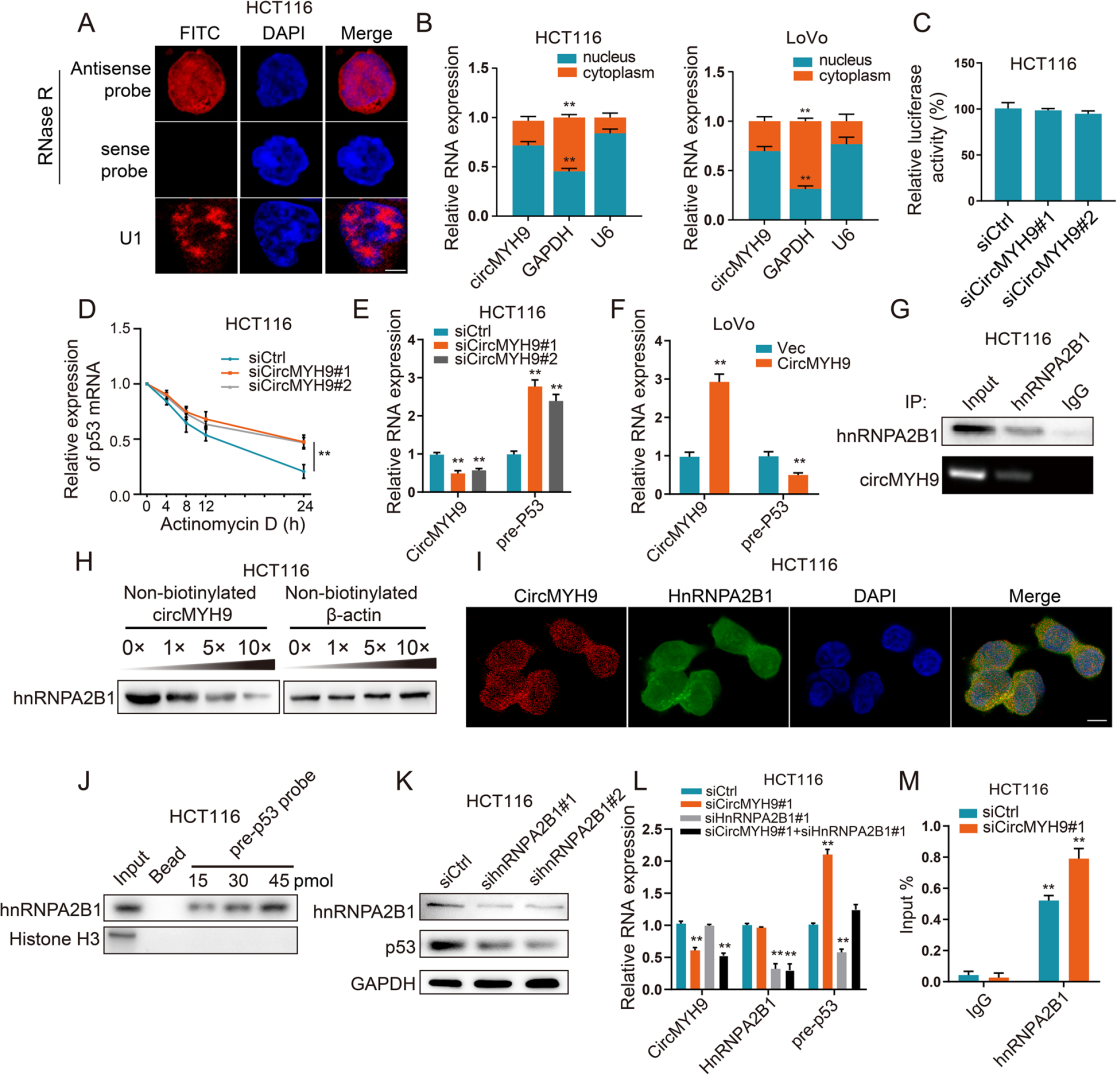
CircMYH9 suppressed the stability of p53 pre-mRNA in a hnRNPA2B1-dependent manner. **A**, RNA-FISH assay showed the nuclear localization of circMYH9 in HCT116 cells incubated with circMYH9 probe (red) with the nuclei staining with DAPI (blue). The U6 were applied as positive controls (red). Scale bar: 5 μm. **B** qRT-PCR indicated the distribution of GAPDH, U6, and circMYH9 in the cytoplasmic and nuclear fractions of CRC cells. **C** Relative luciferase activity of the p53 promoter firefly luciferase reporter in the HCT116 cells transfected with circMYH9 siRNAs or the control. Data are shown as the relative ratio of firefly luciferase activity to the control group. **D** The decay rate of p53 mRNA after treatment with 2.5 μM actinomycin D for the indicated times, with circMYH9 knockdown in HCT116 cells. **E–F** The RNA levels of circMYH9 and p53 pre-mRNA (pre-p53) were analysed by qRT-PCR in the CircMYH9-depleted or circMYH9 overexpressed CRC cells. **G** RIP with an hnRNPA2B1 antibody combined with RT-PCR or immunoblot detection showed the interaction between circMYH9 and hnRNPA2B1 in HCT116 cells. **H** RNA pulldown assay using non-biotinylated probe for circMYH9 combined with IB revealed that specific binding of circMYH9 to hnRNPA2B1 was confirmed by competition assays. Non-biotinylated probe for β-actin was used as control. **I** Colocalization analysis was performed with specific probes against circMYH9 (red) and a specific antibody against hnRNPA2B1 (green). Nuclei are stained with DAPI. Scale bar: 5 μm. **J** RNA pulldown assay using gradient concentration of p53 pre-mRNA probe combined with IB revealed specific binding of p53 pre-mRNA to hnRNPA2B1. **K** IB analysis of hnRNPA2B1 and p53 expression in the hnRNPA2B1-silenced HCT116 cells and control HCT116 cells. **L** qRT-PCR examined the expression of circMYH9, hnRNPA2B1 and p53 pre-mRNA in the HCT116 cells treated with circMYH9 siRNA and/or hnRNPA2B1 siRNA. **M** RIP combined with qRT-PCR using an antibody specific for hnRNPA2B1 detecting the expression of p53 pre-mRNA in the circMYH9-depleted HCT116 cells. Data are shown as the mean ± SD of three independent experiments (*, *P* < 0.05; **, *P* < 0.01)

To explain the mechanism by which circMYH9 regulates p53 expression, we performed qRT-PCR and FISH to determine the subcellular location of circMYH9 in HCT116 and LoVo cells. The results showed that circMYH9 was mainly localized in the nucleus (Fig. 4A, B). As circMYH9 knockdown did not affect p53 promoter activity, as shown by a promoter reporter assay (Fig. 4C), we assessed the stability of p53 mRNA. Interestingly, after blocking new RNA synthesis with actinomycin D, we found that silencing circMYH9 did not alter p53 mRNA stability within 12 h but significantly enhanced p53 mRNA stability at 24 h (Fig. 4D), suggesting that circMYH9 might indirectly inhibit the mRNA degradation of p53. Indeed, p53 pre-mRNA expression was increased following circMYH9 siRNA treatment and decreased following circMYH9 plasmid treatment (Fig. 4E, F). The results suggested that circMYH9 depletion might lead to an increase in p53 pre-mRNA and subsequently affect the mRNA expression of p53.

Binding to specific protein(s) is an important strategy for circRNAs to implement their functions. Therefore, we verified whether certain proteins participate in regulating the stability of p53 pre-mRNA by circMYH9. ChIRP combined with MS was performed to identify the binding proteins of circMYH9, and 42 proteins, including hnRNPA2B1, were identified in this process (Table S2). HnRNPA2B1 is a nuclear N6-methyladenosine (m^6^A) reader which binds to nascent RNA and thus affects a perplexing array of RNA metabolism exquisitely [19]. To validate the MS results, we performed an RIP assay and observed enrichment of circMYH9 in complexes precipitated by an hnRNPA2B1 antibody (Fig. 4G, Figure S4A). The interaction between hnRNPA2B1 and circMYH9 was competitively inhibited by non-biotinylated circMYH9 in a dose-dependent manner (Fig. 4H). Immunofluorescence colocalization assays showed that circMYH9 and hnRNPB2A1 colocalized in the nucleus in CRC cells (Fig. 4I), pearson's correlation analysis (Rx) and correlation coefficient analysis (R) showed that Rx and R were 0.599 ± 0.053 and 0.610 ± 0.045 respectively, which highlight a high degree of correlation.

To examine the association of hnRNPA2B1 and p53 pre-mRNA, The RIP assay using anti-hnRNPA2B1 antibody was performed, demonstrating that hnRNPA2B1 interacts with p53 pre-mRNA (Figure S4B). We pulled down p53 pre-mRNA using a biotinylated antisense DNA probe. hnRNPA2B1 was coprecipitated (Fig. 4J, Figure S4C). hnRNPA2B1 knockdown led to a decrease in the pre-mRNA and protein levels of p53 (Fig. 4K, Figure S4D). These results suggested that hnRNPA2B1 may exert stabilizing effects on p53 pre-mRNA. We further evaluate whether hnRNPA2B1 can bridge circMYH9 and p53 pre-mRNA, the qPCR results showed that hnRNPA2B1 knockdown could eliminate the promoting effects of circMYH9 depletion on the expression of p53 pre-mRNA (Fig. 4L), whereas, hnRNPA2B1 overexpression could rescue the downregulated expression of p53 pre-mRNA induced by circMYH9 overexpression (Figure S4E). We next evaluated whether circMYH9 affects the binding of hnRNPA2B1 to p53 pre-mRNA. The results revealed that circMYH9 knockdown increases the binding between hnRNPA2B1 and p53 pre-mRNA (Fig. 4M, Figure S4F), whereas increased circMYH9 expression attenuated hnRNPA2B1 binding to p53 pre-mRNA (Figure S4G). These findings suggest that circMYH9 suppresses the stability of p53 pre-mRNA by prevent hnRNPA2B1 binding to p53 pre-mRNA.

hnRNPA2B1 includes two RNA recognition motifs (RRM1 and RRM2), which have been reported to be putative RNA-binding domains. To identify the binding domain for hnRNPA2B1, we generated three recombinant hnRNPA2B1s with Flag tags and transfected them into HCT116 cells (Figure S4H). RIP assays with Flag antibodies and qRT-PCR showed that Flag-hnR2 containing the RRM2 motif could immunoprecipitate circMYH9 and p53 pre-mRNA, whereas Flag-hnR1 and Flag-hnR3 could not (Figure S4I, J). Moreover, as expected, overexpression of hnRNPA2B1 construct containing RRM2 motif promoted the expression of p53 pre-mRNA, whereas the RRM2 motif-deleted hnRNPA2B1 construct lost its inhibitory effect on p53 pre-mRNA (Figure S4K). Taken together, these data demonstrated that the RRM2 motif of hnRNPA2B1 mediates the circMYH9-induced regulation of the stability of p53 pre-mRNA.

### The p53 3' untranslated region (UTR) mediated hnRNPA2B1 regulation of pre-mRNA stability

**Fig. 5:**
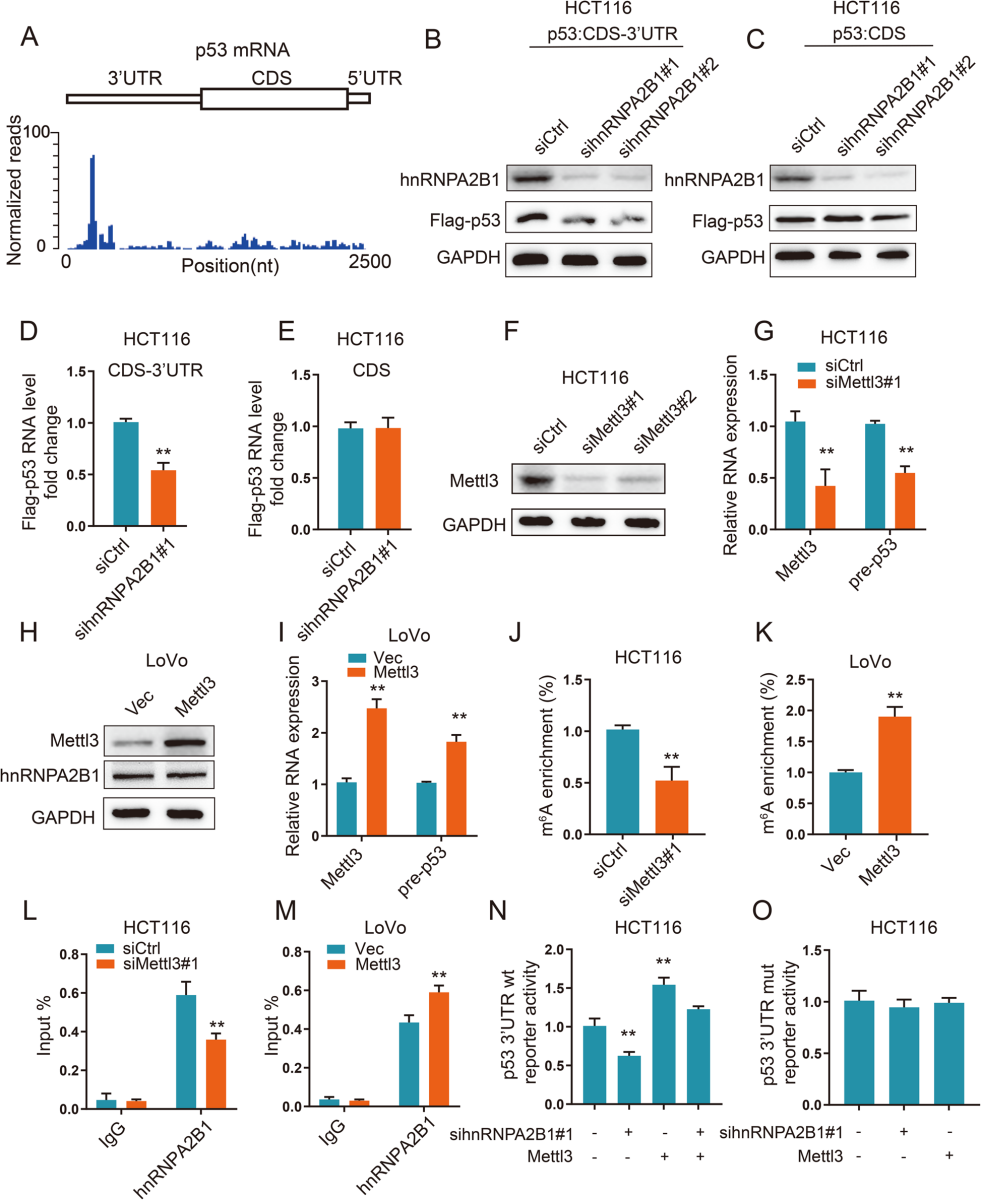
The p53 3' UTR mediated hnRNPA2B1 regulation of pre-mRNA stability. **A** MeRIP-seq data in HCT116 cells showed the distribution of m^6^A peaks along p53 mRNA. One significant m^6^A peak (chr17:7,571,893–7,572,013) was distributed in the 3'UTR region of p53 mRNA. **B-C** IB analysis of Flag-p53 levels in the HCT116 cells expressing Flag-p53 with the p53 CDS-3'UTR or p53 CDS and treated with control or hnRNPA2B1 siRNAs. **D-E** qRT-PCR analysis of Flag-p53 levels in the HCT116 cells expressing Flag-p53 with the p53 CDS-3'UTR or p53 CDS and treated with control or hnRNPA2B1 siRNAs. The sequence of the forward primer corresponds to the 3 × Flag vector. **F** IB analysis of Mettl3 levels in the HCT116 cells transfected with Mettl3 siRNAs. **G** qRT-PCR analysis of the expression of p53 pre-mRNA in the HCT116 cells transfected with Mettl3 siRNAs compared to those transfected with the siRNA control. **H** IB analysis of Mettl3 and hnRNPA2B1 levels in the LoVo cells transfected with Mettl3 plasmid. **I** qRT-PCR analysis of the expression of p53 pre-mRNA in the LoVo cells transfected with Mettl3 plasmid compared to those transfected with the vector. **J-K** MeRIP-qPCR analysis of m^6^A enrichment of p53 3'UTR in the CRC cells transfected with Mettl3 siRNA or overexpression plasmid compared to the cells transfected with the siRNA control or empty vector. **L-M** RIP analysis of the interaction of hnRNPA2B1 with p53 pre-mRNA in the CRC cells transfected with Mettl3 siRNA or overexpression plasmid compared to the cells transfected with the siRNA control or empty vector. **N–O** Relative activity of the wild-type or mutant p53 3'UTR firefly luciferase reporter in the HCT116 cells cotransfected with control or hnRNPA2B1 siRNAs and empty vector or Mettl3 overexpression plasmid. Data are shown as the mean ± SD of three independent experiments (*, *P* < 0.05; **, *P* < 0.01)

To further determine which region of p53 participates in regulation of stability, we performed MeRIP-seq in HCT116 cells and found that one significant m^6^A peak (chr17:7,571,893–7,572,013) was distributed in the 3'UTR of p53 mRNA (Fig. 5A). We constructed a Flag-tagged expression vector including the p53 coding sequence with the 3'UTR (p53 CDS-3'UTR) or the CDS alone (p53 CDS) and cotransfected HCT116 cells with hnRNPA2B1 siRNA. The results showed that hnRNPA2B1 knockdown significantly decreased the expression of p53 in the CRC cells transfected with the p53 CDS-3'UTR rather than the p53 CDS alone (Fig. 5B-E). suggesting that the 3'UTR was indispensable for the hnRNPA2B1-mediated regulation of p53.

hnRNPA2B1 has also been regarded as the "reader" of m^6^A and can stabilize transcripts [19]. We evaluated whether m^6^A methylation was involved in the regulatory effects of hnRNPA2B1 on p53 pre-mRNA. Mettl3 was demonstrated to serve as the primary methyltransferase critical for m^6^A methylation [20]. Our results revealed that depletion of the m^6^A writer Mettl3 decreased the p53 pre-mRNA levels in HCT116 cells (Fig. 5F, G), whereas the overexpression of Mettl3 increased p53 pre-mRNA levels without affecting the protein level of hnRNPA2B1 (Fig. 5H, I). MeRIP-qPCR with specific primers for the p53 3'UTR and CDS region was performed to detect the m^6^A level, and the results showed that silencing Mettl3 led to decreased levels of p53 m^6^A methylation in the 3'UTR peak (Fig. 5J), while overexpressing Mettl3 had the opposite effect (Fig. 5K). Next, RIP assays confirmed that the interaction between hnRNPA2B1 and p53 pre-mRNA was suppressed by depleting Mettl3 or enhanced by overexpressing Mettl3 (Fig. 5L, M).

To further confirm that hnRNPA2B1 increases p53 pre-mRNA by binding to its m^6^A sites, we conducted p53 3'UTR luciferase reporter assays. We found that hnRNPA2B1 knockdown decreased the activity of the luciferase construct containing the p53 3'UTR, and Mettl3 overexpression increased the reduced luciferase activity induced by hnRNPA2B1 knockdown (Fig. 5N). Next, the adenine in the m^6^A consensus sequence in the p53 3'UTR was mutated (5'-AAACT-3' to 5'-AAUCT-3'), and a p53 3'UTR mut luciferase assay was established. We found that the luciferase activity of p53 3'UTR mut showed no response upon hnRNPA2B1 knockdown or Mettl3 overexpression (Fig. 5O). These data revealed that the expression of p53 pre-mRNA was controlled by hnRNPA2B1 binding to m^6^A sites on the p53 3'UTR.

### CircMYH9 was activated by the ROS-HIF1α pathway under amino acid deprivation

**Fig. 6:**
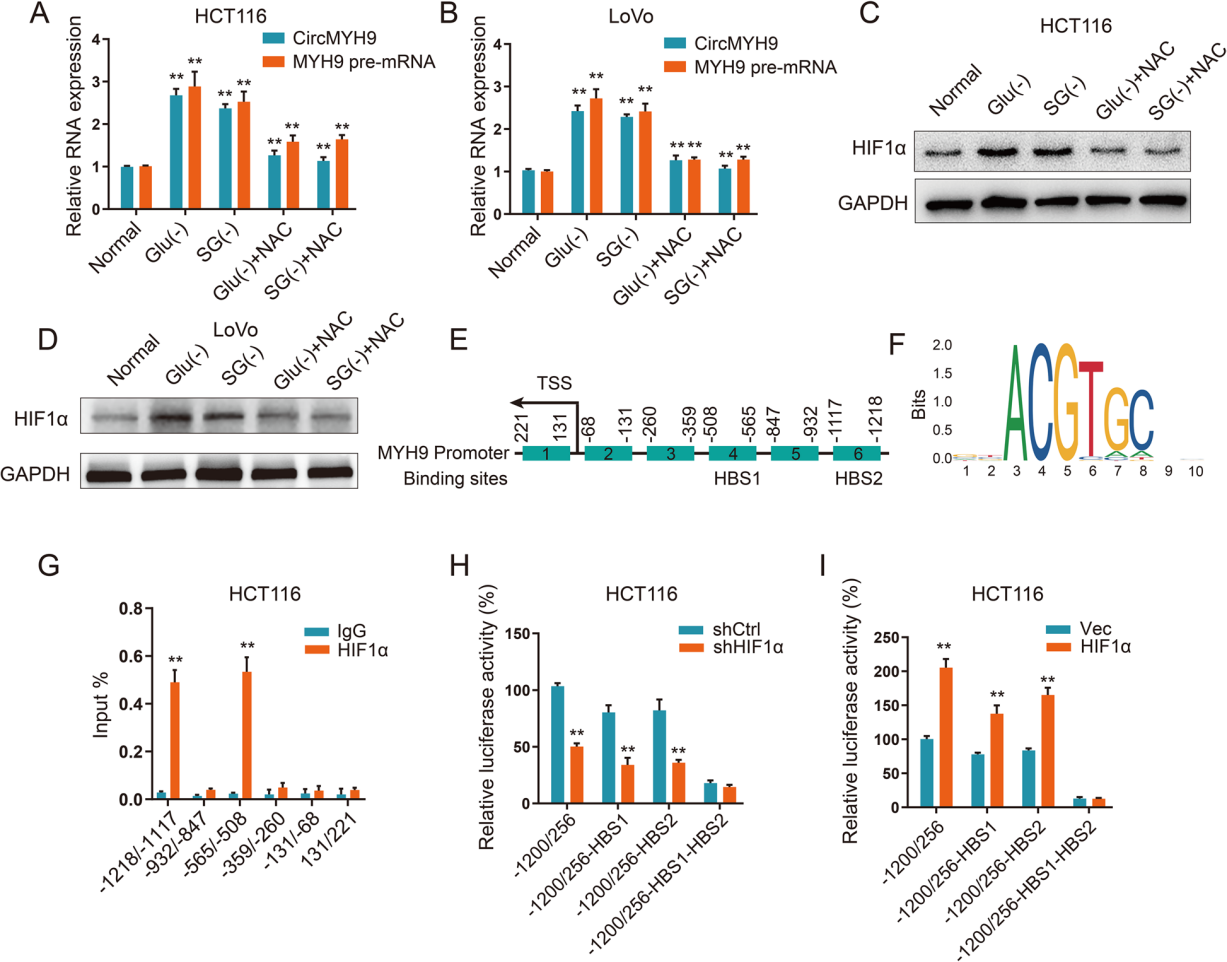
CircMYH9 was activated by the ROS-HIF1α pathway under amino acid deprivation. **A-B** The levels of circMYH9 and MYH9 pre-mRNA were determined by qRT-PCR in CRC cells cultured in complete, SG-free or Glu-free medium with or without NAC treatment. **C-D** The levels of HIF1α were determined by IB in complete, SG-free or Glu-free medium with or without NAC treatment. **E** A schematic of the six MYH9 promoter regions (1–6) analysed for HIF1α binding affinity. HBS1 and HBS2 referred to as HIF1α binding sites. **F** The HIF1α-binding motif enriched in the MYH9 promoter was predicted by JASPAR. **G** ChIP-qPCR analysis was used to determine the binding affinity of HIF1α to six MYH9 promoter regions in HCT116 cells, showing that p53 bound to HBS1(-1218/-1117) and HBS2(-565/-508) in the MYH9 promoter. ChIP-qPCR with IgG was performed as the control. **H-I** Luciferase reporter gene constructs were cotransfected with HIF1α shRNA or HIF1α overexpression plasmid in HCT116 cells, and reporter gene activity was measured after 48 h by a dual luciferase assay. The relative value in HCT116 cells cotransfected with shCtrl or vector was set to 100%. Data are shown as the mean ± SD of three independent experiments (*, *P* < 0.05; **, *P* < 0.01)

Cancer cells might transiently or permanently become auxotrophic for nonessential or semi-essential amino acids, and we detected whether amino acid deprivation is related to circMYH9 overexpression in CRC cells. We cultured CRC cells in medium either lacking SG or lacking glutamine (Glu), and circMYH9 was upregulated in response to SG or Glu deprivation (Fig. 6A, B).

It has been reported that Glu or SG deprivation can lead to elevated levels of ROS [21], which can result in an increase in HIF1α [22]. Accordingly, we used the ROS scavenger NAC to treat SG- or Glu-deprived cells. As expected, NAC reversed the elevation of HIF1α and circMYH9 in SG- or Glu-free medium (Fig. 6A-D), furthermore, HIF1α knockout eliminated the Glu or SG deprivation-induced circMYH9 expression in HCT116 and LoVo cells (Figure S5A-D). Suggesting that cellular ROS accumulation induced by SG or Glu deprivation increased the HIF1α levels, thereby promoting circMYH9 expression.

To explore the molecular mechanism of HIF1α promotion of circMYH9 expression, we measured the expression of MYH9 pre-mRNA. SG or Glu starvation increased the pre-mRNA levels of MYH9 (Fig. 6A, B), HIF1α knockdown decreased the MYH9 pre-mRNA levels in the SG- or Glu-deprived CRC cells (Figure S5A-D). These results suggest that HIF1α promotes MYH9 pre-mRNA at the transcriptional level, which further increases circMYH9. HIF1α can activate the transcription of a gene by binding to the consensus hypoxia response element (HRE) in the promoter region [23]. We thus searched for HIF1α binding sites and motifs in the MYH9 promoter using JASPAR (Fig. 6E, F). We identified the two most likely HIF1α binding sites (HBS1 and HBS2): -508 bp to -565 bp and -1117 bp to -1218 bp. ChIP-qPCR demonstrated that HIF1α bound to the two HBS sites of the MYH9 promoter in HCT116 cells (Fig. 6G). To confirm that HIF1α promotes MYH9 expression via HBSs, we constructed luciferase reporters containing the full-length MYH9 promoter region (ranging from -1200 to + 256) and the HBS-deleted circMYH9 promoter region. Deletion of HBS1 or HBS2 partly abolished the inhibition or promotion of MYH9 luciferase reporter activity by HIF1α knockdown or overexpression, while deletion of HBS1 and HBS2 completely abolished the effects of HIF1α shRNA or plasmid on MYH9 luciferase reporter activity (Fig. 6H, I). These data suggest that HBS regions are essential for HIF1α-dependent regulation of circMYH9 transcription under SG or Glu deprivation.

### CircMYH9 promoted colorectal tumorigenesis in p53 wild-type mice

**Fig. 7:**
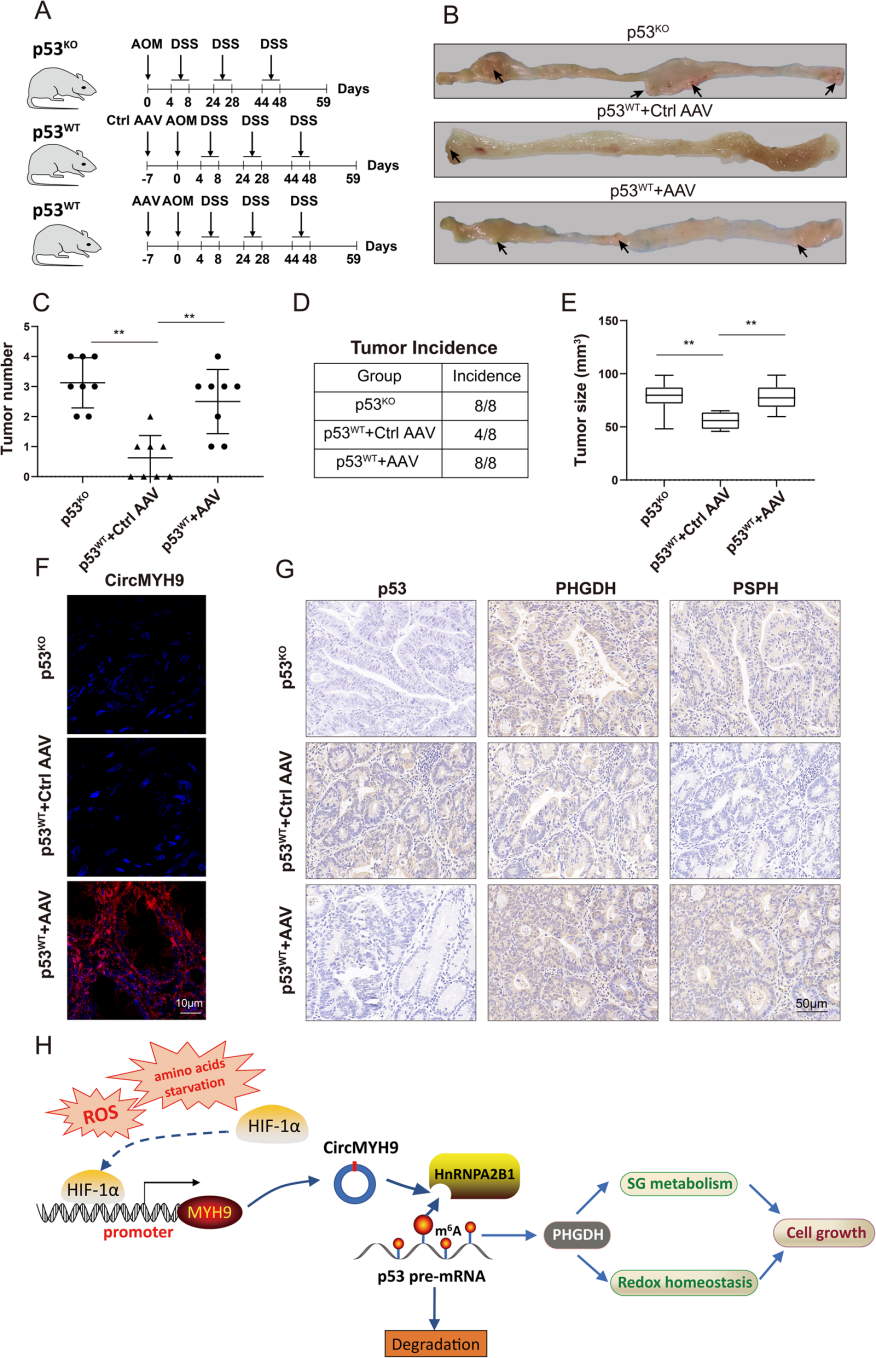
CircMYH9 promoted colorectal tumorigenesis in p53 wild-type mice. **A** Scheme for the AOM/DSS-induced colon cancer model in p53^WT^ and p53^KO^ mice. One week before AOM injection, AAV9-circMYH9 and AAV-control were administered by enema. **B** Representative images of the visible tumors (arrows) in the p53^KO^, p53^WT^ + Ctrl AAV and p53^WT^ + AAV groups. **C-E** The tumour number, tumor incidence and tumor size in each group were analysed, data are shown as the mean ± SD (*, *P* < 0.05; **, *P* < 0.01). **F** The expression level of circMYH9 was detected by FISH in sections of tumours from each group. **G** The expression of p53, PHGDH and PSPH were detected by IHC in sections of tumours from each group. **H** Schematic representation showing that circMYH9 was amplified by amino acid starvation though the ROS-HIF1α pathway. circMYH9 destabilized the pre-mRNA of p53 by preventing hnRNPA2B1 binding to m^6^A sites on the 3' UTR of p53 pre-mRNA. Downregulation of p53 increased the expression of PHGDH, which maintained SG metabolism and redox homeostasis and promoted the growth and tumorigenesis of CRC

To identify a causal role for circMYH9 in CRC pathogenesis in vivo, we generated colon-specific, conditional p53^KO^ mice by crossing p53^fl/fl^ mice with Villin-Cre mice. As shown in Fig. 7 A, p53^WT^ mice and p53^KO^ mice were treated with azoxymethane (AOM) and dextran sodium sulphate (DSS) for 59 days to induce colorectal tumorigenesis. One week before AOM injection, we used AAV9 as a vehicle to specifically overexpress circMYH9 in the colon of mice (1 × 10^11^vp/mouse). The transfection efficiency of AAV-circMYH9 was validated by qPCR (Figure S5E). As expected, p53^KO^ mice developed more numbers, incidence and size of tumors compared to p53^WT^ mice, overexpression of circMYH9 in p53^WT^ mice led to an increase in number, incidence and size of tumors compared to that of the p53^wt^ mice treated with AAV-ctrl (Fig. 7B-E). FISH with a probe against circMYH9 confirmed that circMYH9 was still expressed in tumor tissues of p53^wt^ + AAV mice (Fig. 7F, Figure S5F). IHC examination revealed that the tumors with circMYH9 overexpression showed suppressed expression of p53 and increased expression of PHGDH and PSPH, compared to those in the ctrl-AAV mice (Fig. 7G, Figure S5G). Taken together, these results demonstrated that circMYH9 can drive chemically-induced carcinogenesis by suppressing p53 and promoting SG metabolism in mice.

## Discussion

Most circRNAs are produced from exons, and a small portion are produced from introns. Intronic circRNA is derived from intronic lariats during exon skipping and undergoes internal backsplicing corresponding to a lariat without a tail [24]. The first intronic circRNA was identified in 2013 [25] but has rarely been studied in mammalian tissues to date. In our current study, we identified an intronic circRNA, circMYH9, which was significantly upregulated in CRC tissues and could promote the proliferation and cell cycle of CRC cells. High circMYH9 expression was associated with worse clinical characteristics, p53 expression and poor survival of CRC patients. This finding identifies potential biomarkers for predicting CRC prognosis and provides potential therapeutic targets for CRC treatment, especially for CRC without p53 mutation.

MSI is a hypermutable phenotype caused by the loss of DNA mismatch repair activity. CRC patients with MSI have a slightly better prognosis than CRC patients without MSI and do not have the same response to chemotherapeutics [26]. In our study, MSI was detected in 11 of 145 colorectal cancers, and the incidence of MSI-H was 7.6%, which was similar to that seen in other MSI studies carried out in Asia [27, 28] but was lower than the incidence of MSI-H in a Western population (approximately 15%) [26]. The analysis of the relationship between MSI and circMYH9 expression showed no statistical significance; because there were fewer patients with MSI-H, more patients should be assessed in further research.

To elucidate the mechanism resulting in tumour growth, we performed RNA-seq to evaluate the altered gene expression caused by circMYH9 depletion. Interestingly, the differentially expressed genes were enriched in the pathway related to SG metabolism. The cytosolic synthesis of serine in many cancer cells appears to be in excess of that needed to support macromolecular synthesis [29], and these observations indicated that SG metabolism contributed to tumorigenesis and cancer progression [17]. Serine and glycine synthetic enzymes include PHGDH, PSAT1, PSPH, SLC1A4 and SHMT. Among these, PHGDH was the first rate-limiting enzyme that diverted the glycolytic intermediate 3-phosphoglycerate to serine synthesis [30]. Accordingly, our data demonstrated that circMYH9 can promote the expression of serine and glycine synthetic enzymes to facilitate serine and glycine biosynthesis and sustain tumour growth. Recent studies have demonstrated that the oxidation of serine was required to generate NADPH to suppress ROS and maintain the ratio of reduced to oxidized GSH [31], suggesting that the alteration of SG metabolism is linked with changes in redox status. Intriguingly, we confirmed that the suppressed proliferative capacities induced by circMYH9 depletion result, at least in part, from perturbations of their redox state, which manifested as decreased levels of GSH and the NAD + /NADH ratio and increased levels of ROS.

Our results also demonstrated that p53 was involved in the regulatory roles of circMYH9 in SG metabolism and the redox state. Wild type p53 functions as a classic tumour suppressor and can prevent malignant transformation by controlling genomic integrity and inducing cell death, cell cycle arrest, or cellular senescence [32]. However, approximately half of all CRCs show TP53 gene mutations, and mutant p53 lacks the tumor-suppressive activity [33]. In our study, we found that circMYH9 could significantly promote cell proliferation in p53wt cells, while similar effects were not observed in p53 mutant cells, suggesting that circMYH9 regulates cell proliferation depending on wt p53. Growing evidence has demonstrated that p53 contributes to the metabolic reprogramming of cancer cells. For example, p53 can inhibit glycolysis in cancer cells by regulating the transcription of genes including TIGAR and PFKFB4 [34, 35]. p53 promotes pyruvate oxidation in mitochondria by downregulating PDK2 [36]. In addition to its role in suppressing glycolysis and activating oxidative phosphorylation [37], p53 is involved in the regulation of SG metabolism [18, 38]. p53 suppresses PHGDH expression and inhibits de novo serine biosynthesis in melanoma cells cultured in complete medium [18]. In our current study, we demonstrated that PHGDH was the functional target of p53 in CRC cells, which was consistent with previous publications.

To clarify the mechanism by which circMYH9 regulates p53, we performed RNA pulldown combined with MS using a circMYH9 biotinylated probe. Intriguingly, hnRNPA2B1 was pulled down in this process. Furthermore, RIP assays found that hnRNPA2B1 directly interacted with p53 pre-mRNA. This result suggests that hnRNPA2B1 may mediate the regulation of circMYH9 on p53. hnRNPA2B1 is an abundant nuclear RNA binding protein (RBP) and is known for its regulatory roles in RNA splicing, pre-RNA processing, DNA repair and genome stability. Moreover, hnRNPA2B1 functions as a m^6^A binding protein and could selectively bind to m6A-containing transcripts via the “m^6^A-switch” [39]. The functions of hnRNPA2B1 have not been well documented. It was reported that hnRNPA2B1 regulates expression of Lin28B via binding to Lin28B mRNA and enhancing its stability, thus promoting malignant capability of ovarian cancer [40]. In our study, we noted that hnRNPA2B1 bound to the m^6^A modification sites on the 3'UTR of p53 pre-mRNA, and hnRNPA2B1 knockdown significantly decreased the stability and expression of p53 pre-mRNA (Fig. 7H). In fact, this study is the first to demonstrate that hnRNPA2B1 can modulate m^6^A modification-mediated pre-mRNA stability.

As cancer cells might transiently or permanently become auxotrophic for amino acids [41], we eliminated SG or Glu in the medium and found that amino acid deprivation induced circMYH9 expression. Studies have shown that SG or Glu is required for the synthesis of GSH, which can eliminate excessive ROS [42]. Additionally, ROS can activate HIF1α by inhibiting prolyl hydroxylases (PHDs) to stabilize HIF1α [43]. In our study, we used the ROS inhibitor NAC to treat SG- or Glu-deprived cells, and the elevation of HIF1α and circMYH9 was reversed. Silencing HIF1α could also mimic the effects of NAC. The ROS/HIF1α pathway is involved in the elevation of circMYH9 induced by amino acid deprivation (Fig. 7H). Furthermore, our work identified a negative feedback loop between redox homeostasis and circMYH9 expression. Studies have suggested that the deprivation of specific amino acids, such as SG and Glu, is a promising anticancer strategy with the potential to slow tumour progression [44]. As the elevation of circMYH9 under amino acid deprivation can compromise the antitumour effects of amino acid deprivation, the combination of amino acid deprivation with pharmacological depletion of circMYH9 might offer a promising strategy for enhancing the response to cancer therapy.

## Conclusions

Our study demonstrated for the first time that intron-derived circMYH9 promotes the growth of CRC by modulating SG metabolism and redox homeostasis. We also identified p53 as the target of circMYH9 and showed that circMYH9 can inhibit p53 by degrading p53 pre-mRNA with recruitment of the m^6^A reader hnRNPA2B1. p53 further transcriptionally represses PHGDH expression. Moreover, a ROS/HIF1α pathway that amplifies circMYH9 was identified under SG or Glu starvation. Our work highlights a novel mechanism of circMYH9 in CRC, which provides a valuable resource for monitoring and treating CRC.

## Supplementary Information

**Additional file 1: Figure S1**. **A**, The list of the overlapping upregulated circRNAs. **B**, The panel indicating the detection of circMYH9 and its parent linear mRNA in HCT116 cells treated with or without RNase R (3U/mg). **C**, the quantification of immunofluorescence of circMYH9 expression in peritumour tissues and CRC tissues. **D**, qRT-PCR showed the expression of circMYH9 in normal intestinal epithelial cell line FHC and CRC cell lines. **E**, qRT-PCR examined the expression of circMYH9 after knockdown in CRC cells. **F**, The quantification of colony formation in control HCT116 cells and circMYH9-depleted HCT116 cells. **G-H**, CCK-8 and colony formation assays were applied to detect cell viability of circMYH9-depleted HCT8 cells. **I**, qRT-PCR examined the expression of circMYH9 after overexpression in CRC cells. **J**, The quantification of colony formation in control LoVo cells and circMYH9-overexpressing LoVo cells. **K-L**, CCK-8 assays detected cell viability of circMYH9-depleted DLD1 and HT-29 cells. **M-O**, Flow cytometry was used to assess the cell cycle distribution of control CRC cells and circMYH9-depleted CRC cells. **P**, Cell cycle proteins were assessed by IB in circMYH9-depleted HCT8 cells. **Q**, The histogram shows the quantification of the cell cycle in circMYH9-overexpressing LoVo cells. **R-S**, The quantification of Ki67 IHC staining in sections obtained from xenograft models from each group. Data are shown as the mean ± SD from three independent experiments (*, *P* < 0.05; **, *P* <0.01).

**Additional file 2: Figure S2**. **A-B**, The heatmap showing the expression of genes involved in serine/glycine metabolism and the p53 pathway. **C**, The levels of SG biosynthetic pathway genes were detected by qRT-PCR in the control or circMYH9-overexpressing LoVo cells treated with or without Nutlin-3. **D**, Proliferation was detected by CCK-8 assays in control or circMYH9-overexpressing LoVo cells treated with or without Nutlin-3. **E**, The levels of SG biosynthetic pathway genes were detected by qRT-PCR in the control or circMYH9-depleted HCT116 cells treated with or without p53 shRNA. **F**, Proliferation was detected by CCK-8 assays in control or circMYH9-depleted HCT116 cells treated with or without p53 shRNA.** G**, The levels of SG biosynthetic pathway genes were detected by qRT-PCR in the control or circMYH9-overexpressing LoVo cells treated with or without PHGDH shRNA. **H**, Proliferation was detected by CCK-8 assays in control or circMYH9-overexpressing LoVo cells treated with or without PHGDH shRNA. **I-J**, The quantification of IHC staining of p53, PHGDH and PSPH in xenograft tumours transfected with circMYH9-depleted or circMYH9-overexpressing CRC cells. **K-L**, The luciferase reporter constructs of PHGDH were treated with p53 shRNA or nutlin-3 in CRC cells, and reporter gene activity was measured after 48 h by a dual luciferase assay. The relative value in CRC cells cotransfected with shCtrl or vector was set to 100%. **M-N**, The luciferase reporter constructs of PHGDH were treated with circMYH9 shRNA or overexpression plasmid in CRC cells, and reporter gene activity was measured after 48 h by a dual luciferase assay. The relative value in control CRC cells was set to 100%. **O-P**, ChIP-qPCR analysis was used to assess the binding affinity of p53 to the PHGDH promoter regions after p53 knockdown or overexpression in CRC cells. Data are shown as the mean ± SD from three independent experiments (*, *P* < 0.05; **, *P* <0.01).

**Additional file 3: Figure S3**. **A**, ROS assay indicating the level of ROS in CRC cells transfected with circMYH9 shRNA or overexpression plasmid. **B**, Flow cytometry using the fluorophore dichlorodihydrofluorescein diacetate (DCFDA) to determine the ROS levels in the CRC cells transfected with circMYH9 shRNA or overexpression plasmid. **C**, Immunofluorescence analysis of ROS in CRC cells transfected with circMYH9 shRNA or overexpression plasmid. **D**, Relative levels of reduced and oxidized glutathione (GSH and GSSG) were examined in CRC cells transfected with circMYH9 shRNA or overexpression plasmid. **E**, The NAD+/NADH ratio was examined in HCT116 cells expressing control or circMYH9 shRNA. **F**, ROS levels were reversed in the circMYH9-overexpressing HCT116 cells treated with Nutlin-3 or PHGDH shRNA. **G**, Proliferation was detected by CCK-8 assays in control or circMYH9-overexpressing HCT116 cells treated with or without NAC. Data are shown as the mean ± SD from three independent experiments (*, *P* < 0.05; **, *P* <0.01).

**Additional file 4: Figure S4**. **A**, RIP with an hnRNPA2B1 antibody combined with RT-PCR or immunoblot detection showed the interaction between circMYH9 and hnRNPA2B1 in LoVo cells. **B**, RIP combined with qRT-PCR using an antibody specific for hnRNPA2B1 showing the interaction between p53 pre-mRNA and hnRNPA2B1 in HCT116 cells. IgG and ACTB were used as control. **C**, RNA pulldown assay using gradient concentration of p53 pre-mRNA probe combined with IB revealed specific binding of p53 pre-mRNA to hnRNPA2B1 in LoVo cells. **D**, qRT-PCR analysis of hnRNPA2B1 and p53 pre-mRNA expression in the hnRNPA2B1-silenced HCT116 cells and control HCT116 cells. **E**, qRT-PCR examined the expression of circMYH9, hnRNPA2B1 and p53 pre-mRNA in the LoVo cells treated with circMYH9 overexpression plasmid and/or hnRNPA2B1 overexpression plasmid. **F-G**, RIP combined with qRT-PCR using an antibody specific for hnRNPA2B1 detecting the expression of p53 pre-mRNA in the circMYH9-depleted or circMYH9 overexpressing CRC cells. **H**, Schematic of the three Flag-HnRNPA2B1 recombinant proteins (HnR1-HnR3). Plasmids encoding a Flag-tagged, hnRNPA2B1 truncation mutant were transfected in CRC cells. **I-J**, qRT-PCR was used to determine the expression of circMYH9 and pre-p53 immunoprecipitated by anti-Flag antibody for recombinant proteins. **K**, Overexpression of hnR-2, but not hnR-1 and hnR-3, increased the RNA expression of pre-p53. Data are shown as the mean ± SD from three independent experiments (*, *P* < 0.05; **, *P* <0.01).

**Additional file 5: Figure S5**. **A-B**, The levels of circMYH9, HIF1α and MYH9 pre-mRNA were determined by IB in CRC cells cultured in SG-free or Glu-free medium with or without HIF1α knockdown. **C-D**, The levels of circMYH9, HIF1α and MYH9 pre-mRNA were determined by qRT-PCR in the CRC cells cultured in SG-free or Glu-free medium with or without HIF1α knockdown. **E**, qRT-PCR detected the transfection efficiency of AAV-circMYH9 and ctrl AAV in colon tissue of mice. **F**, The quantification of immunofluorescence in sections from p53KO, p53WT+Ctrl AAV and p53WT+AAV mice. **G**, The quantification of IHC staining of p53, PHGDH and PSPH in sections of tumours from p53KO, p53WT+Ctrl AAV and p53WT+AAV group. Data are shown as the mean ± SD from three independent experiments (*, *P* < 0.05; **, *P* <0.01).

**Additional file 6: Table S1**.

**Additional file 7: Table S2**.

**Additional file 8**: Supplemental Materials and Methods.

**Additional file 9**: Additional blots for internal reference gene.

## Data Availability

The datasets used and/or analysed during the current study are available from the corresponding author on reasonable request.

## Abbreviations

CRC: Colorectal cancer
ChIP: Chromatin immunoprecipitation
CCK8: Cell Counting Kit-8
ChIRP: Chromatin isolation by RNA purification
GSEA: Gene set enrichment analysis
GO: Gene ontology
ncRNAs: Noncoding RNAs
ROS: Reactive oxygen species
TCGA: The Cancer Genome Atlas
IHC: Immunohistochemistry
qRT-PCR: Quantitative real-time PCR
3PG: 3-Phosphoglycerate
LC–MS: Liquid chromatography-mass spectrometry
GSH: Glutathione
GSSG: Glutathione disulphide
NAC: N-acetylcysteine
UTR: Untranslated region
m^6^A: N6-methyladenosine
Glu: Glutamine
SG: Serine and glycine
FISH: Fluorescence in situ hybridization
IB: Immunoblot
RIP: RNA immunoprecipitation
OS: Overall survival
RFS: Relapse-free survival
