# Supplementary figures and images for: CircMYH9 drives colorectal cancer growth by regulating serine metabolism and redox homeostasis in a p53-dependent manner
Source: Mol Cancer. 2021 Sep 8;20:114. doi: 10.1186/s12943-021-01412-9 (PMC8424912; doi:10.1186/s12943-021-01412-9)

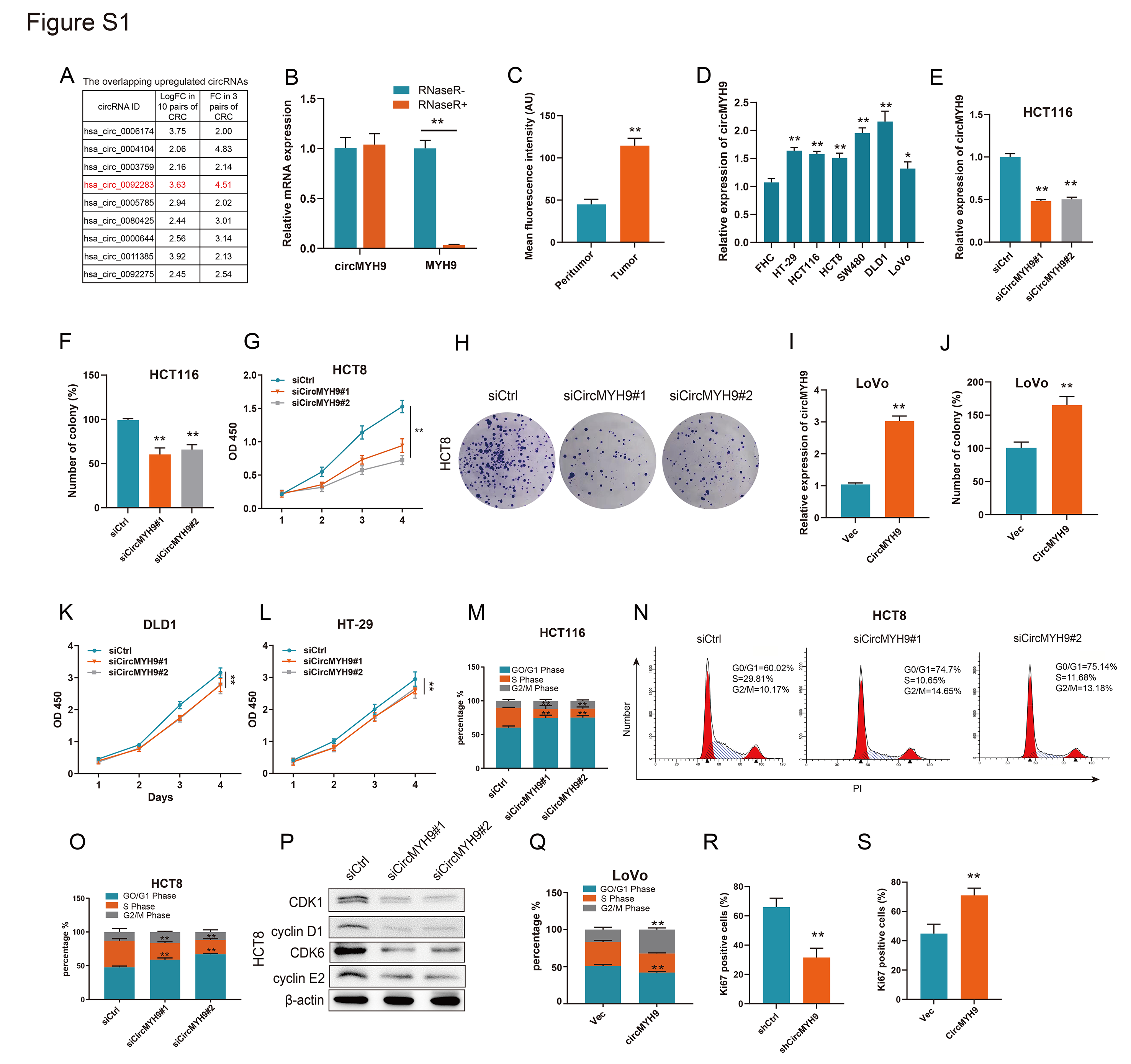

Supplement: Supplementary file 1 — Additional file 1: Figure S1. A, The list of the overlapping upregulated circRNAs. B, The panel indicating the detection of circMYH9 and its parent linear mRNA in HCT116 cells treated with or without RNase R (3U/mg). C, the quantification of immunofluorescence of circMYH9 expression in peritumour tissues and CRC tissues. D, qRT-PCR showed the expression of circMYH9 in normal intestinal epithelial cell line FHC and CRC cell lines. E, qRT-PCR examined the expression of circMYH9 after knockdown in CRC cells. F, The quantification of colony formation in control HCT116 cells and circMYH9-depleted HCT116 cells. G-H, CCK-8 and colony formation assays were applied to detect cell viability of circMYH9-depleted HCT8 cells. I, qRT-PCR examined the expression of circMYH9 after overexpression in CRC cells. J, The quantification of colony formation in control LoVo cells and circMYH9-overexpressing LoVo cells. K-L, CCK-8 assays detected cell viability of circMYH9-depleted DLD1 and HT-29 cells. M-O, Flow cytometry was used to assess the cell cycle distribution of control CRC cells and circMYH9-depleted CRC cells. P, Cell cycle proteins were assessed by IB in circMYH9-depleted HCT8 cells. Q, The histogram shows the quantification of the cell cycle in circMYH9-overexpressing LoVo cells. R-S, The quantification of Ki67 IHC staining in sections obtained from xenograft models from each group. Data are shown as the mean ± SD from three independent experiments (*, P < 0.05; **, P <0.01). [file 12943_2021_1412_MOESM1_ESM.tif]

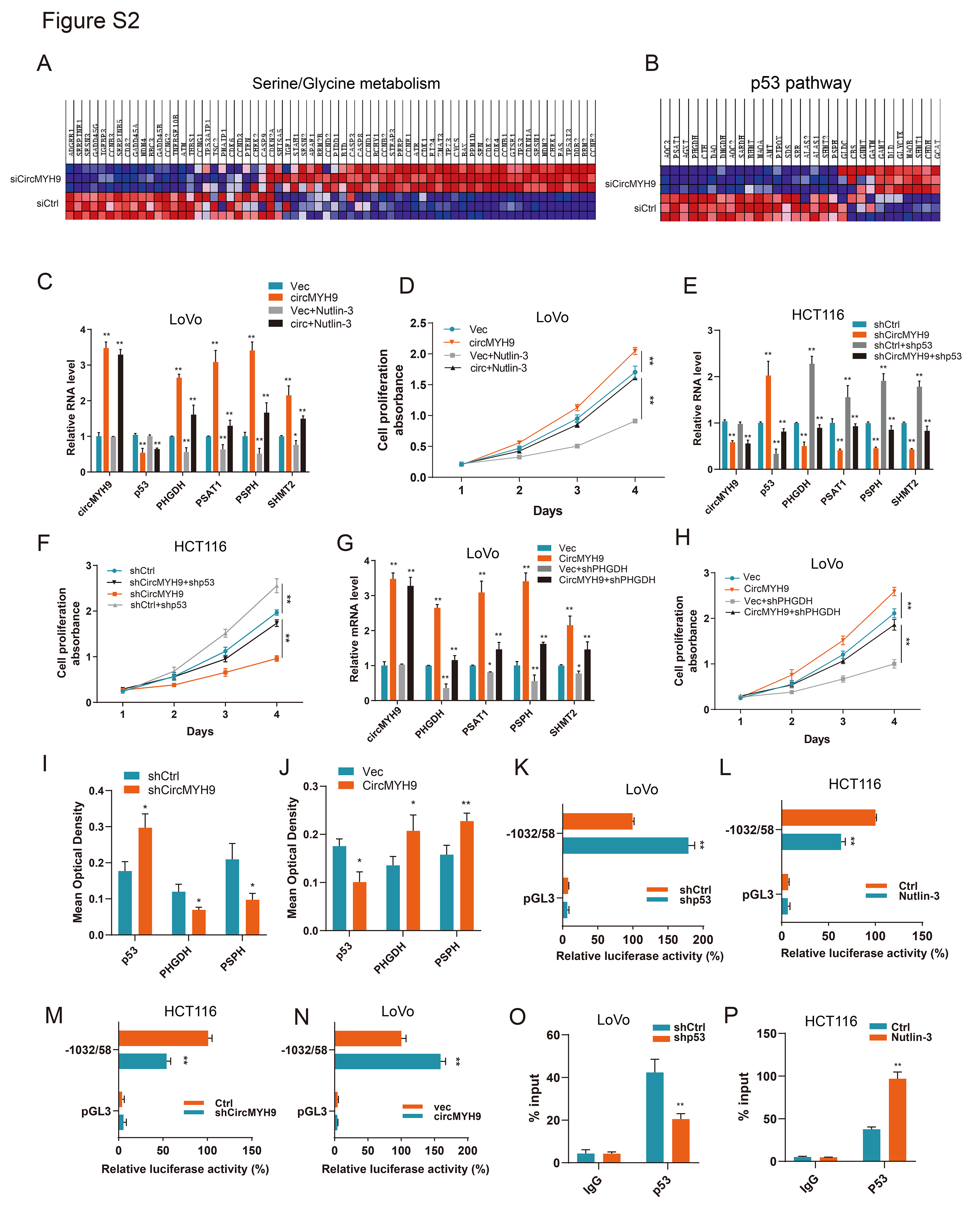

Supplement: Supplementary file 2 — Additional file 2: Figure S2. A-B, The heatmap showing the expression of genes involved in serine/glycine metabolism and the p53 pathway. C, The levels of SG biosynthetic pathway genes were detected by qRT-PCR in the control or circMYH9-overexpressing LoVo cells treated with or without Nutlin-3. D, Proliferation was detected by CCK-8 assays in control or circMYH9-overexpressing LoVo cells treated with or without Nutlin-3. E, The levels of SG biosynthetic pathway genes were detected by qRT-PCR in the control or circMYH9-depleted HCT116 cells treated with or without p53 shRNA. F, Proliferation was detected by CCK-8 assays in control or circMYH9-depleted HCT116 cells treated with or without p53 shRNA. G, The levels of SG biosynthetic pathway genes were detected by qRT-PCR in the control or circMYH9-overexpressing LoVo cells treated with or without PHGDH shRNA. H, Proliferation was detected by CCK-8 assays in control or circMYH9-overexpressing LoVo cells treated with or without PHGDH shRNA. I-J, The quantification of IHC staining of p53, PHGDH and PSPH in xenograft tumours transfected with circMYH9-depleted or circMYH9-overexpressing CRC cells. K-L, The luciferase reporter constructs of PHGDH were treated with p53 shRNA or nutlin-3 in CRC cells, and reporter gene activity was measured after 48 h by a dual luciferase assay. The relative value in CRC cells cotransfected with shCtrl or vector was set to 100%. M-N, The luciferase reporter constructs of PHGDH were treated with circMYH9 shRNA or overexpression plasmid in CRC cells, and reporter gene activity was measured after 48 h by a dual luciferase assay. The relative value in control CRC cells was set to 100%. O-P, ChIP-qPCR analysis was used to assess the binding affinity of p53 to the PHGDH promoter regions after p53 knockdown or overexpression in CRC cells. Data are shown as the mean ± SD from three independent experiments (*, P < 0.05; **, P <0.01). [file 12943_2021_1412_MOESM2_ESM.tif]

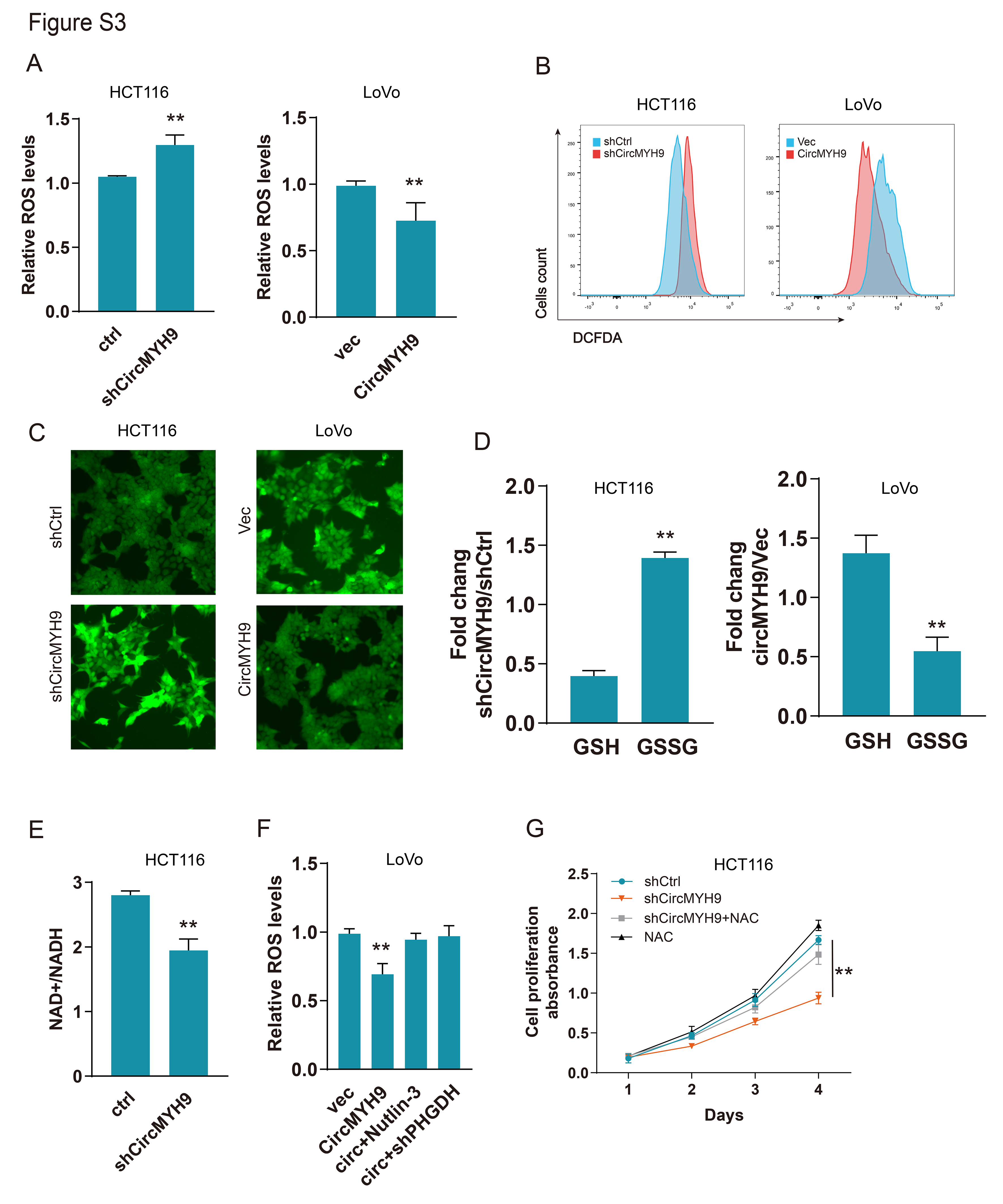

Supplement: Supplementary file 3 — Additional file 3: Figure S3. A, ROS assay indicating the level of ROS in CRC cells transfected with circMYH9 shRNA or overexpression plasmid. B, Flow cytometry using the fluorophore dichlorodihydrofluorescein diacetate (DCFDA) to determine the ROS levels in the CRC cells transfected with circMYH9 shRNA or overexpression plasmid. C, Immunofluorescence analysis of ROS in CRC cells transfected with circMYH9 shRNA or overexpression plasmid. D, Relative levels of reduced and oxidized glutathione (GSH and GSSG) were examined in CRC cells transfected with circMYH9 shRNA or overexpression plasmid. E, The NAD+/NADH ratio was examined in HCT116 cells expressing control or circMYH9 shRNA. F, ROS levels were reversed in the circMYH9-overexpressing HCT116 cells treated with Nutlin-3 or PHGDH shRNA. G, Proliferation was detected by CCK-8 assays in control or circMYH9-overexpressing HCT116 cells treated with or without NAC. Data are shown as the mean ± SD from three independent experiments (*, P < 0.05; **, P <0.01). [file 12943_2021_1412_MOESM3_ESM.tif]

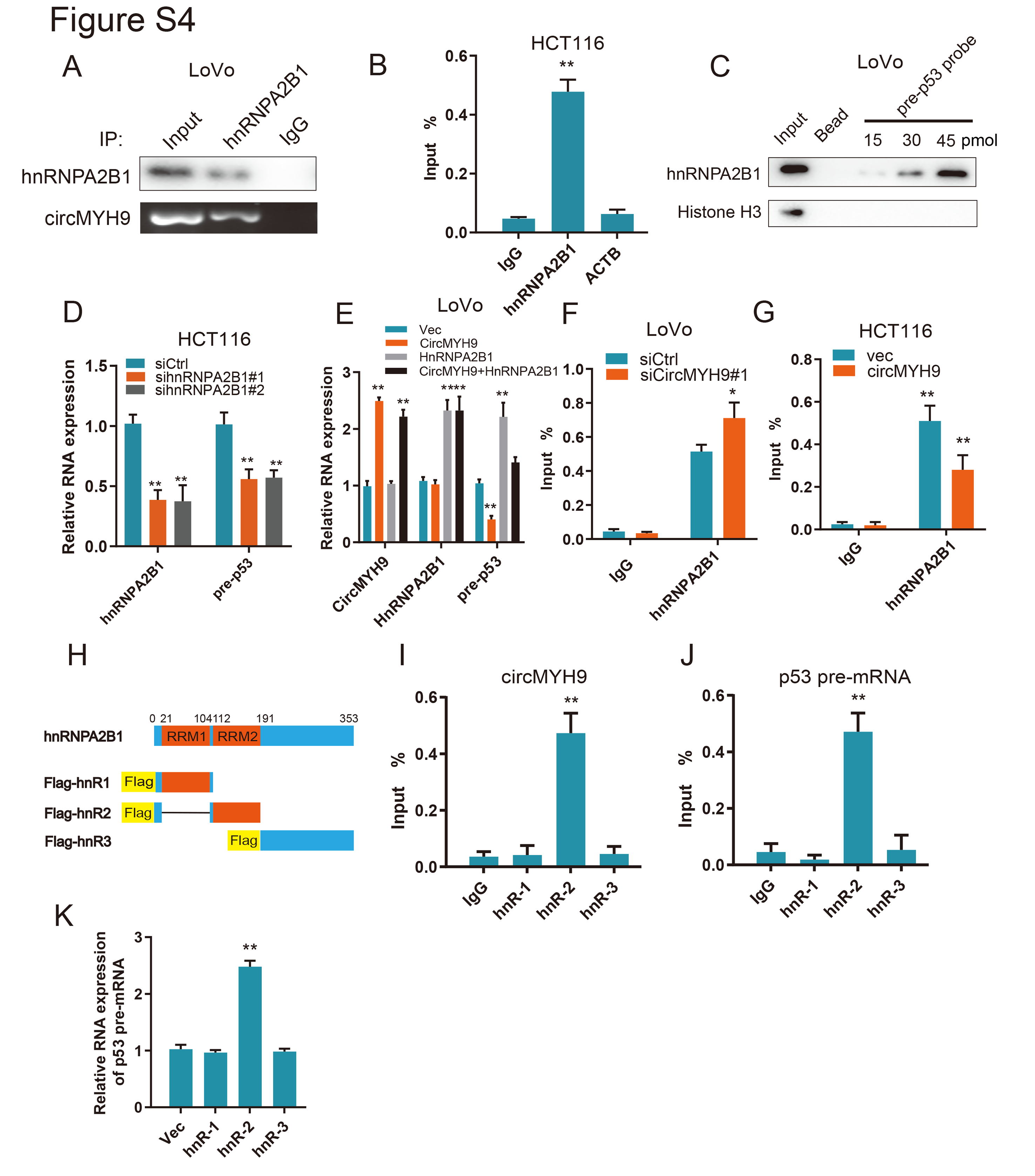

Supplement: Supplementary file 4 — Additional file 4: Figure S4. A, RIP with an hnRNPA2B1 antibody combined with RT-PCR or immunoblot detection showed the interaction between circMYH9 and hnRNPA2B1 in LoVo cells. B, RIP combined with qRT-PCR using an antibody specific for hnRNPA2B1 showing the interaction between p53 pre-mRNA and hnRNPA2B1 in HCT116 cells. IgG and ACTB were used as control. C, RNA pulldown assay using gradient concentration of p53 pre-mRNA probe combined with IB revealed specific binding of p53 pre-mRNA to hnRNPA2B1 in LoVo cells. D, qRT-PCR analysis of hnRNPA2B1 and p53 pre-mRNA expression in the hnRNPA2B1-silenced HCT116 cells and control HCT116 cells. E, qRT-PCR examined the expression of circMYH9, hnRNPA2B1 and p53 pre-mRNA in the LoVo cells treated with circMYH9 overexpression plasmid and/or hnRNPA2B1 overexpression plasmid. F-G, RIP combined with qRT-PCR using an antibody specific for hnRNPA2B1 detecting the expression of p53 pre-mRNA in the circMYH9-depleted or circMYH9 overexpressing CRC cells. H, Schematic of the three Flag-HnRNPA2B1 recombinant proteins (HnR1-HnR3). Plasmids encoding a Flag-tagged, hnRNPA2B1 truncation mutant were transfected in CRC cells. I-J, qRT-PCR was used to determine the expression of circMYH9 and pre-p53 immunoprecipitated by anti-Flag antibody for recombinant proteins. K, Overexpression of hnR-2, but not hnR-1 and hnR-3, increased the RNA expression of pre-p53. Data are shown as the mean ± SD from three independent experiments (*, P < 0.05; **, P <0.01). [file 12943_2021_1412_MOESM4_ESM.tif]

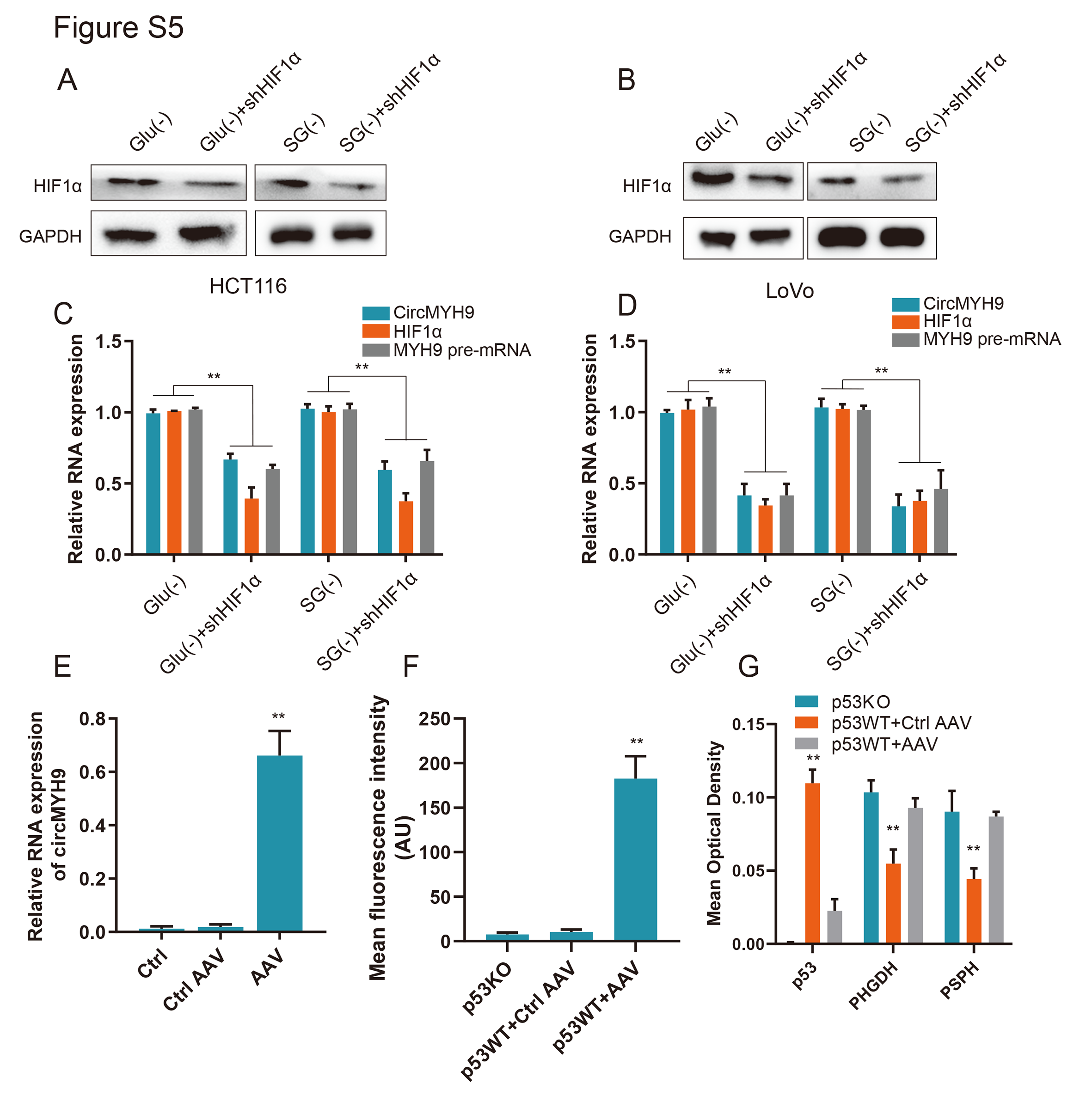

Supplement: Supplementary file 5 — Additional file 5: Figure S5. A-B, The levels of circMYH9, HIF1α and MYH9 pre-mRNA were determined by IB in CRC cells cultured in SG-free or Glu-free medium with or without HIF1α knockdown. C-D, The levels of circMYH9, HIF1α and MYH9 pre-mRNA were determined by qRT-PCR in the CRC cells cultured in SG-free or Glu-free medium with or without HIF1α knockdown. E, qRT-PCR detected the transfection efficiency of AAV-circMYH9 and ctrl AAV in colon tissue of mice. F, The quantification of immunofluorescence in sections from p53KO, p53WT+Ctrl AAV and p53WT+AAV mice. G, The quantification of IHC staining of p53, PHGDH and PSPH in sections of tumours from p53KO, p53WT+Ctrl AAV and p53WT+AAV group. Data are shown as the mean ± SD from three independent experiments (*, P < 0.05; **, P <0.01). [file 12943_2021_1412_MOESM5_ESM.tif]

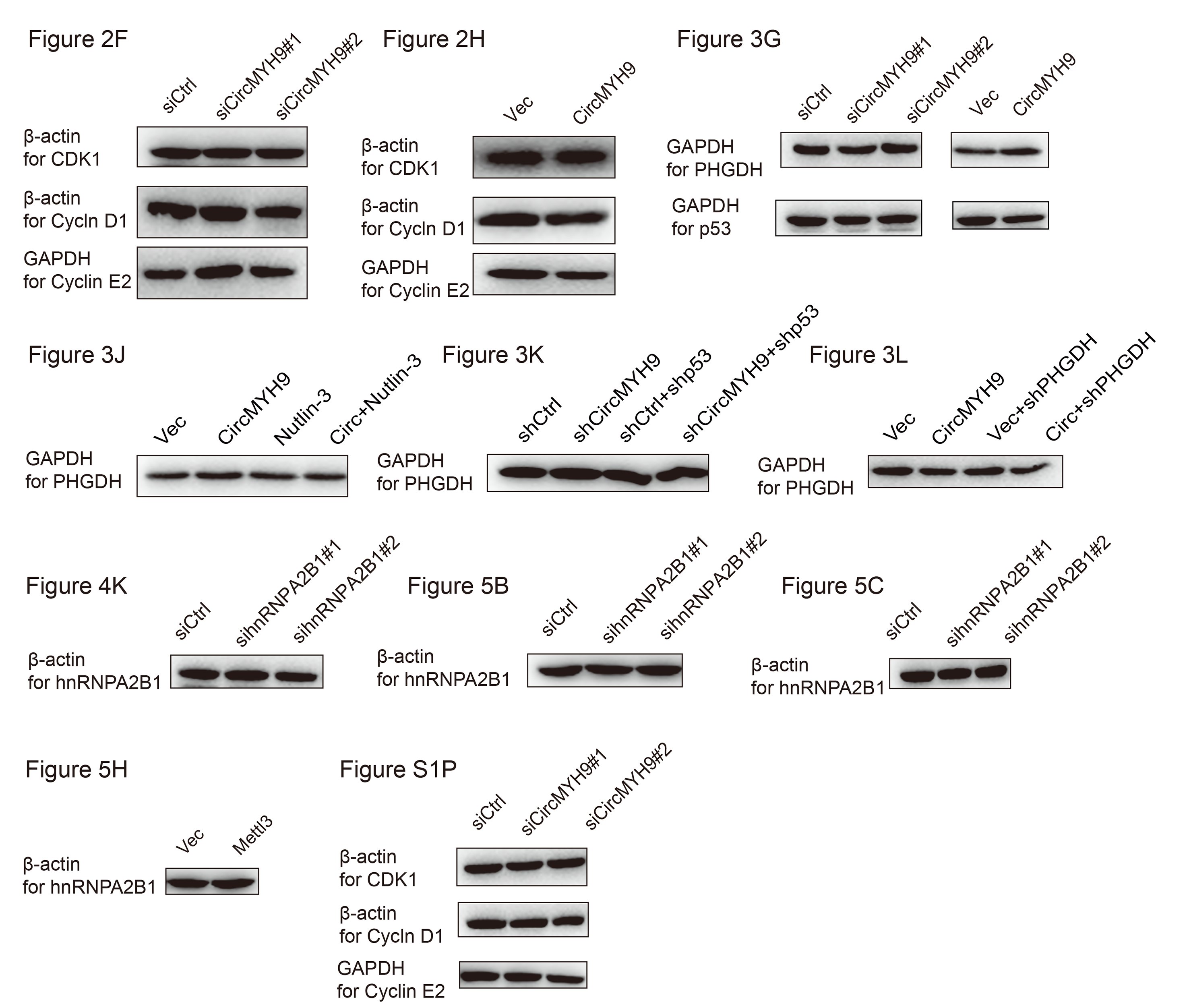

Supplement: Supplementary file 9 — Additional file 9: Additional blots for internal reference gene. [file 12943_2021_1412_MOESM9_ESM.tif]
