# Supplementary material for: CircMYH9 drives colorectal cancer growth by regulating serine metabolism and redox homeostasis in a p53-dependent manner
Source: Mol Cancer. 2021 Sep 8;20:114. doi: 10.1186/s12943-021-01412-9 (PMC8424912; doi:10.1186/s12943-021-01412-9)
Supplement: Supplementary file 6 — Additional file 6: Table S1. [file 12943_2021_1412_MOESM6_ESM.docx]

|  | Tumor size | TNM stage | Lymph node metastasis | Differentiation | MSI status | P53 status |
| --- | --- | --- | --- | --- | --- | --- |
| Patient 1 | >5.0cm | III | positive | Poor | MSI-H | P53(+) |
| Patient 2 | <5.0cm | III | positive | moderate | MSS | P53(+) |
| Patient 3 | >5.0cm | III | positive | moderate | MSS | P53(-) |
