# Supplementary material for: CircMYH9 drives colorectal cancer growth by regulating serine metabolism and redox homeostasis in a p53-dependent manner
Source: Mol Cancer. 2021 Sep 8;20:114. doi: 10.1186/s12943-021-01412-9 (PMC8424912; doi:10.1186/s12943-021-01412-9)
Supplement: Supplementary file 8 — Additional file 8: Supplemental Materials and Methods. [file 12943_2021_1412_MOESM8_ESM.docx]

**Supplemental Materials and Methods**

**Immunoblot (IB)**
Tissues or cells were homogenized and lysed with lysis buffer (50 mM Tris-HCl, 137mM NaCl, 10% glycerol, 100 mM sodium orthovanadate, 1mM phenylmethylsulfonyl fluoride (PMSF), 10 mg/ml aprotinin, 10 mg/ml leupeptin, 1% NP-40, and 5 mM protease inhibitor cocktail; pH 7.4). After protein concentration determination using a bicinchoninic acid (BCA) assay, β-mercaptoethanol and bromophenol blue were added to the sample buffer for electrophoresis. The proteins were separated via 10% PAGE and transferred to polyvinylidene difluoride membranes (Bio-Rad, Shanghai, China). The membranes were incubated with primary antibodies overnight at 4°C. After incubation with secondary antibodies for 1h, the reactive bands were visualized using an enhanced chemiluminescence system. The intensities of the bands were semi-quantified using ImageJ software (v1.53e) according to the instructions.

**Fluorescence in situ hybridization (FISH)**

FISH assay was carried out according to manufacturer’s protocol. Briefly, cells were rinsed in PBS and then fixed in 4% formaldehyde for 10 min at room temperature. Cells were permeabilized in PBS containing 0.5% Triton X-100 for 5 min at 4 °C, then washed in PBS 3 × 5 min. Hybridization was carried out with a FISH probe in a moist chamber at 37 °C in the dark overnight. FISH kit and Specific probes targeting circMYH9, p53 pre-mRNA were purchased from Guangzhou RiboBio Co., Ltd. (China). After RNA FISH, all images were obtained with a Zeiss LSM800 confocal microscope.

**Chromatin immunoprecipitation (ChIP)**A ChIP assay was carried out using an EZ-ChIP^TM^ Chromatin Immunoprecipitation Kit (Millipore, Bedford, MA, USA) following the manufacturer's protocols. Briefly, 1% formaldehyde was used to crosslink proteins and DNA for 10min. Cell lysates were sonicated to obtain DNA fragments, which were subjected to IP with primary antibodies or negative control IgG. Purified DNA was analyzed by qRT-PCR with SYBR Green Master Mix (Cwbio, NanJing, China). The relative enrichment values were calculated through normalization of the results to the input values and are expressed relative to the values obtained with normal IgG. The primers used are described in Supplemental Materials and Methods.

**Stable isotope tracing experiments**

Measurement of glucose-derived 13C incorporation in serine and glycine were performed as previously described(1). Briefly, cells were plated in 6-well plates and cultured in McCoys medium or 1640 medium for 24h before replacing the medium with fresh medium containing 13C-labelled glucose and incubated for the indicated times. Cells were washed with PBS followed by metabolite extraction with ice-cold extraction buffer consisting of methanol, acetonitrile, and H_2_O (50:30:20). Extracts were analyzed by LCMS using a Dionex Ultimate 3000 LC system coupled to a Q Exactive mass spectrometer (Thermo Scientific) as previously described(2). Serine and glycine levels were quantified using five-point calibration curves spiked in cell lysates and media. Metabolites were identified and analyzed using Thermo LCquan software.

**Colony formation**Transfected cells or stable cells were seeded into 6-well plate (5×102/well). The cultures were maintained at 37 °C in a 5%CO_2_ incubator for 2 weeks. The colonies were fixed with 4% paraformaldehyde and stained with 0.5% crystal violet. The numbers of the colony were counted under a microscope.

**Intracellular ROS, Glutathione and NAD+/NADH measurement**

Total intracellular ROS levels were evaluated by immunofluorescence staining with the CM-H2DFCDA probe (Invitrogen, Shanghai, China) or by flow cytometry using a FC500 flow cytometer (Beckman Coulter, USA) upon staining with the CM-H2DFCDA probe according to the manufacturer’s recommendations. The levels of reduced (GSH) and oxidized glutathione (GSSG) were measured with the GSH/GSSG Glo Assay (Promega, Beijing, China) according to the manufacturer’s instructions. The NAD+/NADH ratio was measured in cells using the NAD/NADH Glo Assay (Promega, Beijing, China).

**Dual luciferase reporter assay**

The regions of MYH9 promoter, PHGDH promoter or p53 3’UTR were PCR amplified and cloned into the pGL3 vector (Promega, Madison, WI). Cells were cotransfected with a fireﬂy luciferase reporter expression constructs, a Renilla luciferase expression plasmid (pRL-TK, 20 ng) and shRNA(or overexpression vector). After 48 h, luciferase activity was measured with a Dual Luciferase Reporter Assay System (Promega, Madison, WI).

**Immunohistochemistry (IHC)**

Tissue sections were deparaffinized in xylene and rehydrated using a graded ethanol series. To quench endogenous peroxidase activity, the sections were immersed in a 0.3% peroxidase–methanol solution for 30 minutes. For antigen retrieval, the sections were pretreated with citrate buffer for 15 minutes at 100°C in a microwave oven. The sections were hybridized with a primary antibody at 4°C overnight at a dilution of 1:100 and were visualized using an UltraVision Quanto Detection System HRP DAB Kit (Thermo Scientific, Shanghai, China) according to the manufacturer’s protocols. The stained sections were counterstained with hematoxylin, and photomicrographs were captured using an Olympus BX51 microscope (Olympus, Tokyo, Japan).

**Cell Counting Kit-8 (CCK8)**

CCK8 analysis was carried out according to the manufacturer’s protocol to determine the regulation of cell proliferation by circMYH9. After 48 hours of transfection, cells were seeded in 96-well plates at a density of 4000 cells per well. The absorbance at 450 nm was measured 1 hour after adding the CCK-8 solution.

**RNA immunoprecipitation (RIP)**

RIP was conducted with the Magna RIP RNA-Binding Protein Immunoprecipitation Kit (Millipore, USA) according to the manufacturer’s instructions. Briefly, magnetic beads coated with 5 μg of specific antibodies were incubated with prepared cell lysates overnight at 4°C. Then, the RNA-protein complexes were washed 6 times and incubated with proteinase K digestion buffer. RNA was finally extracted by phenol-chloroform RNA extraction methods. The relative expression of RNA was determined by qPCR and normalized to the input.

**Primers for qRT-PCR**

| Gene | Forward | Reverse |
| --- | --- | --- |
| circMYH9 | CTCATGCCCTCCAGCCAG | GGTCCAAGGCCAGCTCTG |
| PHGDH | CTGCGGAAAGTGCTCATCAGT | TGGCAGAGCGAACAATAAGGC |
| PSAT1 | TGCCGCACTCAGTGTTGTTAG | GCAATTCCCGCACAAGATTCT |
| PSPH | GAGGACGCGGTGTCAGAAAT | GGTTGCTCTGCTATGAGTCTCT |
| SHMT2 | CCCTTCTGCAACCTCACGAC | TGAGCTTATAGGGCATAGACTCG |
| SLC1A4 | TGTTTGCTCTGGTGTTAGGAGT | CGCCTCGTTGAGGGAATTGAA |
| hnRNPA2B1 | CAGGGTAGTTGAGCCAAAACG | TTCCAGACTGCCTATCGGTAA |
| p53 pre-mRNA | CAGTCAGATCCTAGCGTCGA | CCCAACCCTTGTCCTTACCA |
| p53 3’UTR | GGCCCACTTCACCGTACTAA | GTGGTTTCAAGGCCAGATGT |
| p53 CDS | GTTCCGAGAGCTGAATGAGG | TCTGAGTCAGGCCCTTCTGT |
| U6 | CTCGCTTCGGCAGCACATATACT | ACGCTTCACGAATTTGCGTGTC |
| GAPDH | TGCACCACCAACTGCTTAGC | GGCATGGACTGTGGTCATGAG |

**Primers for ChIP**

| Primer | Forward | Reverse |
| --- | --- | --- |
| MYH9  (-1117/-1218) | AAATGTTGGGATTACAGGCA | ACTCTGTGACAAATTACTATCAGC |
| MYH9  (-847/-932) | CAGGTGCACACAGAGAGATCAA | TTTAGTTGGCACCTTCATCGCC |
| MYH9  (-508/-565) | CAGCAACGTGCGGATCCTA | TTGTGAGCGTCTTGAGTTCG |
| MYH9  (-260/-359) | TCCAGACCTTCCCAGTCGG | ACACAGGGGGAATCCAATCAG |
| MYH9  (-68/-131) | GGATGTTGAATCCCCACCCA | TATTGGCCTGTGGAAAGGGG |
| MYH9  (221/131) | GGAAGGCTAAGCAAGGCTGA | CGAGAGGACTTTCTCGAGCG |
| PHGDH | TGAGAATATGCGGTGTTTGG | GGGTAAATGTGCAAGGCACT |

**shRNA and siRNA sequence**

**circMYH9 siRNA#1**

GCCCUCCCCAAGGUCAGGATT

**circMYH9 siRNA#2**

CCAAGGUCAGGACUUCUCCTT

**circMYH9 shRNA**

CACCGCCCTCCCCAAGGTCAGGATTCGAAAATCCTGACCTTGGGGAGGGC**hnRNPA2B1 siRNA #1**

GCAACAAGGAAGGGCATCTTGGTTA

**hnRNPA2B1 siRNA #2**

CAAGGAAGGGCATCTTGGTTATGAA

**Mettl3 siRNA#1**

GACGAATTATCAATAAACACACT

**Mettl3 siRNA#2**

CGCAAGATTGAGTTATTTGGACG

**PHGDH shRNA**

CACCGCAACAAGGAAGGGCATCTTGGTTACGAATAACCAAGATGCCCTTCCTTGTTGC

**p53 shRNA**

CACCGAAACCACTGGATGGAGAATATTCGAAAATATTCTCCATCCAGTGGTTTC

**The luciferase reporter assay**

The primers used for cloning the indicated promoters are as follows.

| **Gene** | **Forward** | **Reverse** |
| --- | --- | --- |
| MYH9 promoter  (-1200/256) | AGGTGAACAAACAGCGAGGT | GCACTCCACTGCTGCCTTC |
| PHGDH promoter  (-1032/58) | GAGAGCACGTCGGTTATGGG | CTCAAACTCTCCGCGACTCC |

**References**

1. Maddocks ODK, Berkers CR, Mason SM, Zheng L, Blyth K, Gottlieb E, et al. Serine starvation induces stress and p53-dependent metabolic remodelling in cancer cells. Nature. 2012;493(7433):542-6.

2. Labuschagne CF, van den Broek NJ, Mackay GM, Vousden KH, Maddocks OD. Serine, but not glycine, supports one-carbon metabolism and proliferation of cancer cells. Cell reports. 2014;7(4):1248-58.
